# Coordination among frequent genetic variants imparts substance use susceptibility and pathogenesis

**DOI:** 10.3389/fnins.2024.1332419

**Published:** 2024-04-10

**Authors:** Avinash Veerappa, Chittibabu Guda

**Affiliations:** ^1^Department of Genetics, Cell Biology and Anatomy, University of Nebraska Medical Center, Omaha, NE, United States; ^2^Center for Biomedical Informatics Research and Innovation, University of Nebraska Medical Center, Omaha, NE, United States

**Keywords:** substance use disorders, whole exome sequencing, addiction, opioids, alcohol, substance abuse, variants, reward

## Abstract

Determining the key genetic variants is a crucial step to comprehensively understand substance use disorders (SUDs). In this study, utilizing whole exome sequences of five multi-generational pedigrees with SUDs, we used an integrative omics-based approach to uncover candidate genetic variants that impart susceptibility to SUDs and influence addition traits. We identified several SNPs and rare, protein-function altering variants in genes, *GRIA3*, *NCOR1*, and *SHANK1*; compound heterozygous variants in *LNPEP*, *LRP1*, and *TBX2*, that play a significant role in the neurotransmitter-neuropeptide axis, specifically in the dopaminergic circuits. We also noted a greater frequency of heterozygous and recessive variants in genes involved in the structural and functional integrity of synapse receptors, *CHRNA4*, *CNR2*, *GABBR1*, *DRD4*, *NPAS4*, *ADH1B*, *ADH1C*, *OPRM1*, and *GABBR2*. Variant analysis in upstream promoter regions revealed regulatory variants in *NEK9, PRRX1, PRPF4B, CELA2A, RABGEF1*, and *CRBN*, crucial for dopamine regulation. Using family-and pedigree-based data, we identified heterozygous recessive alleles in *LNPEP, LRP1* (4 frameshift deletions), and *TBX2* (2 frameshift deletions) linked to SUDs. GWAS overlap identified several SNPs associated with SUD susceptibility, including rs324420 and rs1229984. Furthermore, miRNA variant analysis revealed notable variants in *mir-548 U* and *mir-532*. Pathway studies identified the presence of extensive coordination among these genetic variants to impart substance use susceptibility and pathogenesis. This study identified variants that were found to be overrepresented among genes of dopaminergic circuits participating in the neurotransmitter-neuropeptide axis, suggesting pleiotropic influences in the development and sustenance of chronic substance use. The presence of a diverse set of haploinsufficient variants in varying frequencies demonstrates the existence of extraordinary coordination among them in attributing risk and modulating severity to SUDs.

## 1 Introduction

Substance use disorders (SUDs) is a combination of incentive salience with habit formation causing binge/intoxication; reward deficits and stress surfeits causing withdrawal/negative affect, and finally compromised executive function caused by preoccupation/anticipation (Koob and Volkow, 2016). These combinatorial features ultimately influence neuroadaptations via the modulation of neurocircuits, synaptic systems and receptor molecules (Koob and Volkow, 2016). Frequent use of substance leads to increased craving caused by dopamine which causes gradual changes in brain circuits to form stronger memory synapses (Koob and Volkow, 2010). These adaptations are naïve and susceptible to pruning in case of abstinence (De Santis et al., 2020). However, continued substance-induced insults sustained due to recollection of stressful life events, or an environmental stimulus intensify the after-effect on brain chemistry, strengthening synapses.

Results from the 2022 National Survey on Drug Use and Health conducted by the Substance Abuse and Mental Health Services Administration (SAMHSA) provided disturbing trends in people aged 12 and above who report using following substances within the past month; an estimated 59.8% used tobacco products, vaped nicotine, used alcohol, or used an illicit drug in the past month (also defined as “current use”), including 48.7% who drank alcohol, 18.1% who used tobacco products, 8.3% who vaped nicotine, and 16.5% who used an illicit drug. 22.0% smoked marijuana in the past year; 3.2% used opioids; and 0.4% from fentanyl use. This study also revealed approximately 48.7 million (17.3%) people aged 12 or older with a SUD (Substance Abuse and Mental Health Services Administration, 2023). This frequency indicates the prevailing cumulative effect of variants involved in the manifestation and pathogenesis of SUDs. Gene variants drive susceptibility beginning from experimentation and bingeing to establishing complete dependency (Edenberg and Foroud, 2013). Genetic variants stimulate motivation at each of these stages to develop stronger synapses leading to progression from the initiation to established use of substance before the development of dependence (Volkow et al., 2019). Besides, studies on the withdrawal trait that involves a slow pruning of neuronal synapses and reward-related brain circuits, diminishing maladaptive motivation to seek and take the drug have been directly correlated with the presence of genetic variants (Koob and Volkow, 2016). Hence, identification of the genetic variant predisposition and determination of the causality is crucial for our understanding of SUDs.

Several Genome-wide association studies (GWAS) have been conducted to investigate genetic susceptibility (Han et al., 2011; Treutlein and Rietschel, 2011; Zuo et al., 2014; Nelson et al., 2016; Minica et al., 2018; Tielbeek et al., 2018; Johnson et al., 2020; Zhou et al., 2020; Guerin et al., 2021). However, their outcomes have often been inconclusive due to the presence of extensive heterogeneity in the population, and a lack of definitive causal associations. Therefore, high-resolution sequencing is required to investigate causal variants for a phenotype as complex as SUDs. Though studies performing whole exome sequencing in SUDs are scant, more studies are required to expand the spectrum of SUDs variants (AshaRani et al., 2021). Therefore, the present study was taken up to identify gene variants by re-assessing the exome sequences of SUDs probands. We performed variant calling and used multi-pronged variant filtering approaches combined with forward genetics evidence to identify the spectrum of variants in genes such as glutamate ionotropic receptor AMPA type subunit 3 (*GRIA3*), nuclear receptor corepressor 1 (*NCOR1*), SH3 and multiple ankyrin repeat domains 1 (*SHANK1*), leucyl and cystinyl aminopeptidase (*LNPEP*), LDL receptor related protein 1 (*LRP1*), and T-box transcription factor 2 (*TBX2*), crucial for neurotransmitter and neuropeptide interactions within dopaminergic pathways. Increased occurrences of heterozygous and recessive variants were observed in genes essential for synapse receptor structure and function, including cholinergic receptor nicotinic alpha 4 subunit (*CHRNA4*), cannabinoid receptor 2 (*CNR2*), gamma-aminobutyric acid type B receptor subunit 1 (*GABBR1*), dopamine receptor D4 (*DRD4*), alcohol dehydrogenase 1B (class I), beta polypeptide (*ADH1B*), alcohol dehydrogenase 1C (class I), gamma polypeptide (*ADH1C*), opioid receptor mu 1 (*OPRM1*), and gamma-aminobutyric acid type B receptor subunit 2 (GABBR2). The discovery of numerous haploinsufficient variants across different frequencies underscores their collective impact on SUDs risk and severity. These variants may collectively influence various addiction phases such as, impulsivity or experience seeking, experimentation, substance metabolism, synapse pruning, maladaptation, reward, memory, tolerance, and withdrawal.

## 2 Methods

### 2.1 Data collection

Whole exome sequence data were directly obtained from the corresponding author of a published study (AshaRani et al., 2021). The subjects with SUDs who used substances such as alcohol, nicotine, and opioids were evaluated for alcohol use disorder (AUD) and opioid use disorder (OUD) using the DSM-5 criteria and for nicotine disorder (ND) using the Fagestrom test. Participants who were unable to read English or had a diagnosed bloodborne disease such as hepatitis B or acquired immunodeficiency syndrome were excluded from participation in the study. The study consisted of five family trios, with each trio comprising one proband and two family members, resulting in a total sample size of 15 (substance users = 5 and non-users/healthy controls =10). Family Trios 1, 3, and 4 included the proband and two parents, while Trios 2 and 5 comprised a proband, one parent, and a sibling. Three of the subjects were diagnosed with AUD (Trios 2, 3 and 5), while the remaining two were diagnosed with OUD (Trios 1 and 4). Supplementary Table S1 presents the socio-demographic features of the trios included in this study. Those subjects with either AUD or OUD together with ND were selected for this study. The severity of addiction was measured using Addiction Severity Index-Lite (McLellan et al., 1980). DNA was extracted from the saliva samples of the study participants and whole exome libraries were prepared and sequenced to achieve about 170x coverage (AshaRani et al., 2021).

### 2.2 Variant discovery

We followed the Genome Analysis Toolkit’s (GATK’s) best practices workflows on short variant discovery to call single nucleotide variants (SNVs) and insertion-deletions (indels).^1^ Germline SNVs and indels were called using the reference genome (GRCh38) and annotated to obtain high-quality variants with a set preferred minimum quality threshold of 30. Local realignment around indels and base call quality score recalibration was performed using the GATK version 4.13 with HaplotypeCaller, CombineGVCF, and GenotypeGVCF workflows. Variants were filtered using the Variant Quality Score Recalibration method. SNVs were annotated by the MQRankSum, HaplotypeScore, QD, FS, MQ, ReadPosRankSum adaptive error model, with (Mills_and_1000G_gold_standard.snps.b38.vcf) as the true positive set. These methods were used to accurately identify and filter SNVs and indels in the genomic data to ensure the reliability of downstream analysis. *Anno*var (Wang et al., 2010) and *Ensembl VEP* (McLaren et al., 2016) were used to annotate the variants from variant call format (VCF) file. Additional VCF manipulations such as VCF merging and shared genotype extractions among cohorts were performed using *bcftools* (Danecek et al., 2021), *vcftools* (Danecek et al., 2011), *bgzip* (Danecek et al., 2021), *tabix* (Danecek et al., 2021), *VcffilterJdk* and *j*var*kit* (Lindenbaum, 2015).

### 2.3 Analysis strategy – variant and annotation filtering

**Figure 1:**
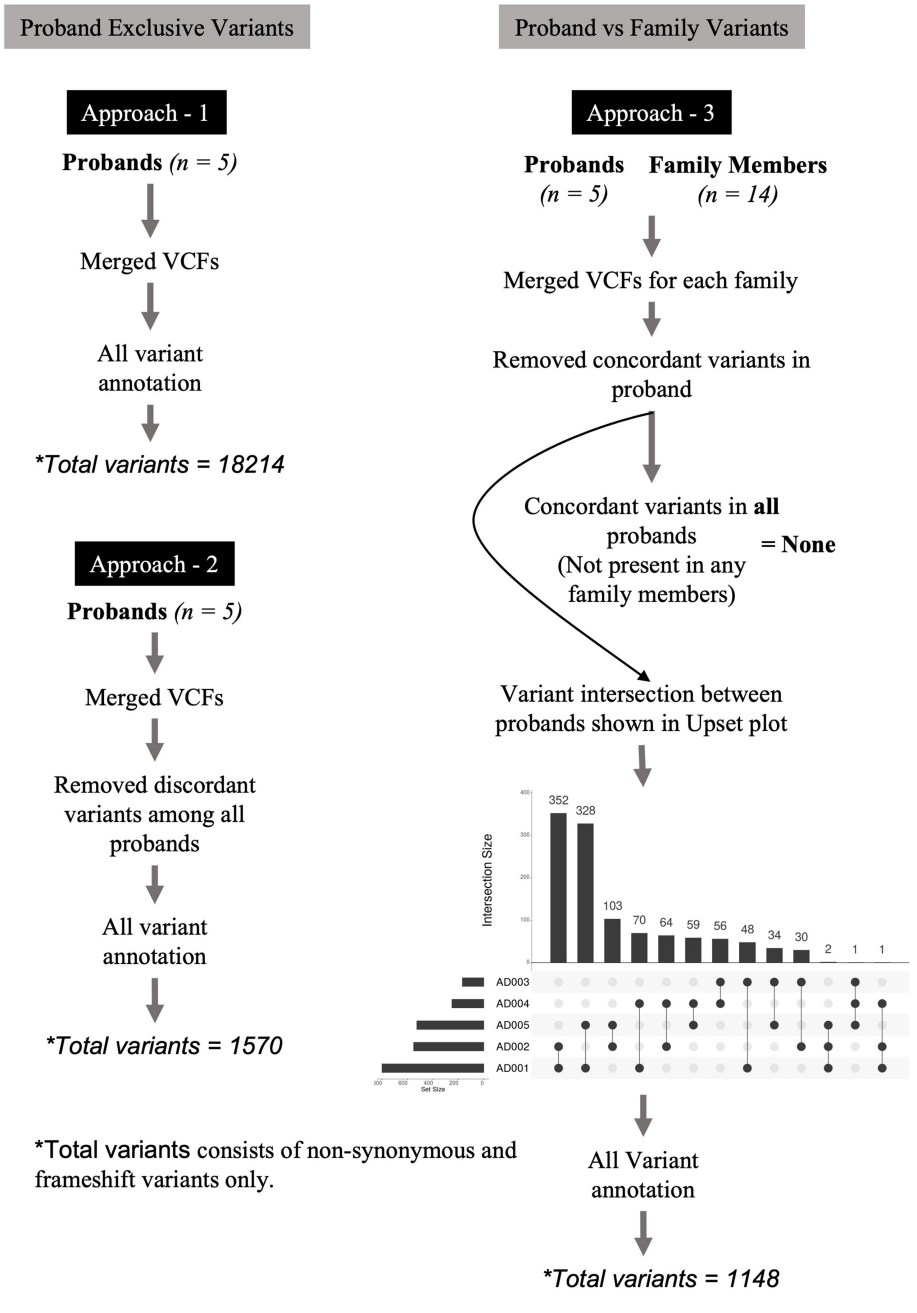
Variant filtering and genotyping was performed using several approaches. In the first approach, the variants of all probands were merged into a multi-sample VCF file and annotated without any variant position filtering. In the second approach, we merged the variants of all probands into a multi-sample VCF file. We applied the vcffilterjdk.jar-e ‘return variant.getGenotype’ feature to remove discordant variants among all probands. The resulting VCF file bearing only the concordant variants across all probands was further used for variant annotation using Annovar and Ensembl VEP. In the third approach, variants in each proband were compared (by position) against variants of corresponding family members. We performed this comparison using ‘bcftools isec -p’ feature and this resulted in the removal of variants in probands that were concordant with their family members to generate VCF files containing only unique variants for the proband. These VCF files were merged into a multi-proband VCF file to identify only concordant variants among them using vcffilterjdk.jar-e ‘return variant.getGenotype’ feature. This filtering approach led us to no variants at the end of the analysis, therefore, we performed variant intersection on pairwise, trio-, quartet-, and quintet comparisons among the probands to build a matrix to identify highest variant concordance among the cohort. Based on the number of shared variants (by position) among probands led us to create a matrix identifying *n* = 3 (subjects AD001, AD002, and AD005) probands that shared higher number of shared variants among themselves. Using this filtering metric, we repeated genotyping in *n* = 3 probands for subjects AD001, AD002, and AD005. This led us to identify two heterozygous nonsynonymous variants in two genes, HRNR and SHANK1. The annotation of variants resulting from all the above three approaches were feature-filtered based on location (exonic), variant type (stop gain, stop loss, nonsynonymous, frameshift deletion, and frameshift insertion), phylo *p* value (≥4) and CADD PHRED score (≥20).

Three approaches were designed to identify relevant variants across varying dimensions. Figure 1 describes these approaches. In the first approach, the variants of all probands were merged into a multi-sample VCF file and annotated without any variant position filtering. This multi-sample VCF file bearing all the variants from probands was then used for variant annotation. The purpose of this approach was to enable the identification of commonly occurring variants [Single Nucleotide Polymorphisms (SNPs)] in SUDs genes. In the second approach, we merged the variants of all probands into a multi-sample VCF file. We then applied the *vcffilterjdk.jar-e ‘return* var*iant.getGenotype - variant.getGenotype.sameGenotype’* feature to remove discordant variants among all probands. In this context, “discordant variants” refer to variants that are not present in all probands. A VCF file containing only the concordant variants (variants referring to those that are present in all probands) identified across all probands was generated. The purpose of this approach was to enable the identification of variants that are unique to cases and absent in controls. In the third approach, variants in each proband were compared (by position) against variants of corresponding family members. We performed this comparison using ‘*bcftools isec-p*’ feature and this resulted in the removal of variants in probands that were concordant (filtering for *de novo* variants) with their family members to generate VCF files containing only unique variants for the proband. The purpose of this approach was to identify *de novo* variants and their role in SUDs. The VCF files were merged into a multi-proband VCF file to identify concordant variants using *vcffilterjdk.jar-e ‘return variant.getGenotype - variant.getGenotype.sameGenotype’* feature. Further, we used ‘*bcftools isec -p*’ feature to identify variant intersection by creating a matrix between probands to identify pairs of probands that share high variant concordance. This VCF file bearing the top 3 concordant variants among the probands (not present in any family members) was further used for variant annotation. VCF files generated from the above three approaches were subjected to annotation using *Annovar* (Wang et al., 2010) and *Ensembl VEP* (McLaren et al., 2016). The annotated variants from all the approaches were feature-filtered based on location (exonic), variant type (stop gain, stop loss, nonsynonymous, frameshift deletion, and frameshift insertion), phylo *p-*value (≥4) and CADD PHRED score (≥20).

### 2.4 Homozygosity mapping

Homozygosity mapping (HM) was performed using the HomozygosityMapper program. The VCFs containing the genotypes were used in HomozygosityMapper to screen all subjects for blocks of homozygous genotypes using contiguous markers. Genome-wide homozygosity was visualized in affected individuals within the chromosomes. HomozygosityMapper reads the length of homozygous blocks in all affected samples for every marker and adds them to a homozygosity score for the respective marker. The homozygous regions were further annotated to identify candidate genes using GeneDistiller, making the process of candidate gene identification faster and easier. To screen for recessive genes and consider them as SUDs genes, certain criteria were applied. The genes had to be implicated by linkage, association, and sequencing studies, and at least one other independently replicated study had to include them. Additionally, the genes should have supported experimental evidence from human and animal studies for their involvement in substance use.

### 2.5 Compound heterozygosity

We used the PhenoDB Variant Analysis Tool (Sobreira et al., 2015) to identify and prioritize the compound heterozygosity (CH) mutations. Because SUDs phenotype is highly variable, and the inheritance pattern being unclear, therefore, it was important to identify the contributions of CH mutations to better understand the disease mechanism.

### 2.6 Promoter variant analysis

The identification of promoter variants is essential for understanding the regulation of gene expression and its impact on SUDs development. Promoter variants alter the binding of TFs, resulting in changes in gene expression levels. By identifying promoter variants, we can identify the contribution of dysregulated gene expression on SUDs development and provides insights into disease pathogenesis. We retrieved data from chromatin immunoprecipitation (ChIP) experiments conducted on several brain regions, including the nucleus accumbens, VTA, dorsolateral prefrontal complex, and midbrain from the Encyclopedia of DNA Elements (ENCODE) project. This data was used to identify regulatory motifs that overlap with the identified variants, and which are bound to transcription factors (TFs).

### 2.7 miRNA variant analysis

The identification of miRNA variants is essential for understanding the role of microRNAs on their mRNA targets and their association with SUDs. Variants in miRNAs or their target sites can alter the expression or function of miRNAs, leading to SUDs pathogenesis. The VCF files generated from the above approaches were subjected to annotation using *Anno*var (Wang et al., 2010) and *Ensembl VEP* (McLaren et al., 2016) to identify the presence of variants in miRNA genes. The annotation was performed based on location, gene symbol, and zygosity with South Asian alleles frequencies derived from 1,000 genome project and the Exome Aggregation Consortium (ExAC). Ingenuity Pathway Analysis was used to map the regulatory axis of the miRNA genes with known SUDs genes.

### 2.8 Functional consequences of DNA variants

Databases such as RegulomeDB (Boyle et al., 2012) and DatabasE of genomiC varIation and Phenotype in Humans using Ensembl Resources (DECIPHER) (pLI > 0.9) (Firth et al., 2009) were used to identify DNA features, regulatory elements, and consequence of variants (haploinsufficiency) to understand their functional consequence. Position Weight Matrix (PWM) scores (bit scores ranging 0–2) were used in regulatory regions to estimate the binding strengths of putative transcription factor binding sites.

### 2.9 Pathway analysis

Pathway analysis on the candidate genes was performed using Ingenuity Pathway Analysis (IPA) (Kramer et al., 2014). IPA was used to map biological processes, canonical pathways, upstream transcriptional regulators, and gene networks. The predicted state, Z-score, and *p*-value enabled us to identify regulators of interest. Downstream Effects Analysis, Causal Network Analysis, and Molecule Activity Predictor (MAP) were performed on the datasets to ascertain the biological significance of genes and proteins. Pathways were constructed by utilizing the *grow and connect* function. Experimental evidence-backed and tissue-specific activations, inhibitions, protein–protein interactions, protein-RNA interactions, and protein-DNA interactions were overlayed on the resulting network to understand the crosstalk among genes. Further, IPA was used to perform upstream regulatory analysis, and mechanistic networks.

## 3 Results

Pedigree-based genome sequence analysis was employed on five family trios to uncover the genetic similarities and dissimilarities and genomic variants were parsed using several hierarchical approaches to identify the SUDs influencing variants. The genotyped variants were annotated for all the three approaches described above and were feature-filtered based on the location (exonic), variant type (stop gain, stop loss, nonsynonymous, frameshift deletion, and frameshift insertion), phylogenetic *p*-values (phyloP) value (≥4) and Combined Annotation Dependent Depletion (CADD) PHRED score (≥20) (Figure 1). To assess the genetic diversity of the variants identified in this study and to categorize them as SNPs (common) and rare variants we compared their allele frequencies to those observed in population-based cohorts, such as the Genome Aggregation Database (gnomAD) and 1,000 genome (1000G) South Asians (SAS).

### 3.1 Genotyping variants in all probands without filtering

In this first approach, the variants of all probands were merged into a multi-sample VCF file and annotated without any variant position filtering. The annotation involved feature-filtering based on location (exonic), variant type (stop gain, stop loss, nonsynonymous, frameshift deletion, and frameshift insertion), phylo *p-*value (≥4) and CADD PHRED score (≥20). Upstream Promoter Analysis, CH variant analysis, HM, and miRNA variant analysis were performed on variants obtained using this approach.

#### 3.1.1 Upstream promoter analysis

**Figure 2:**
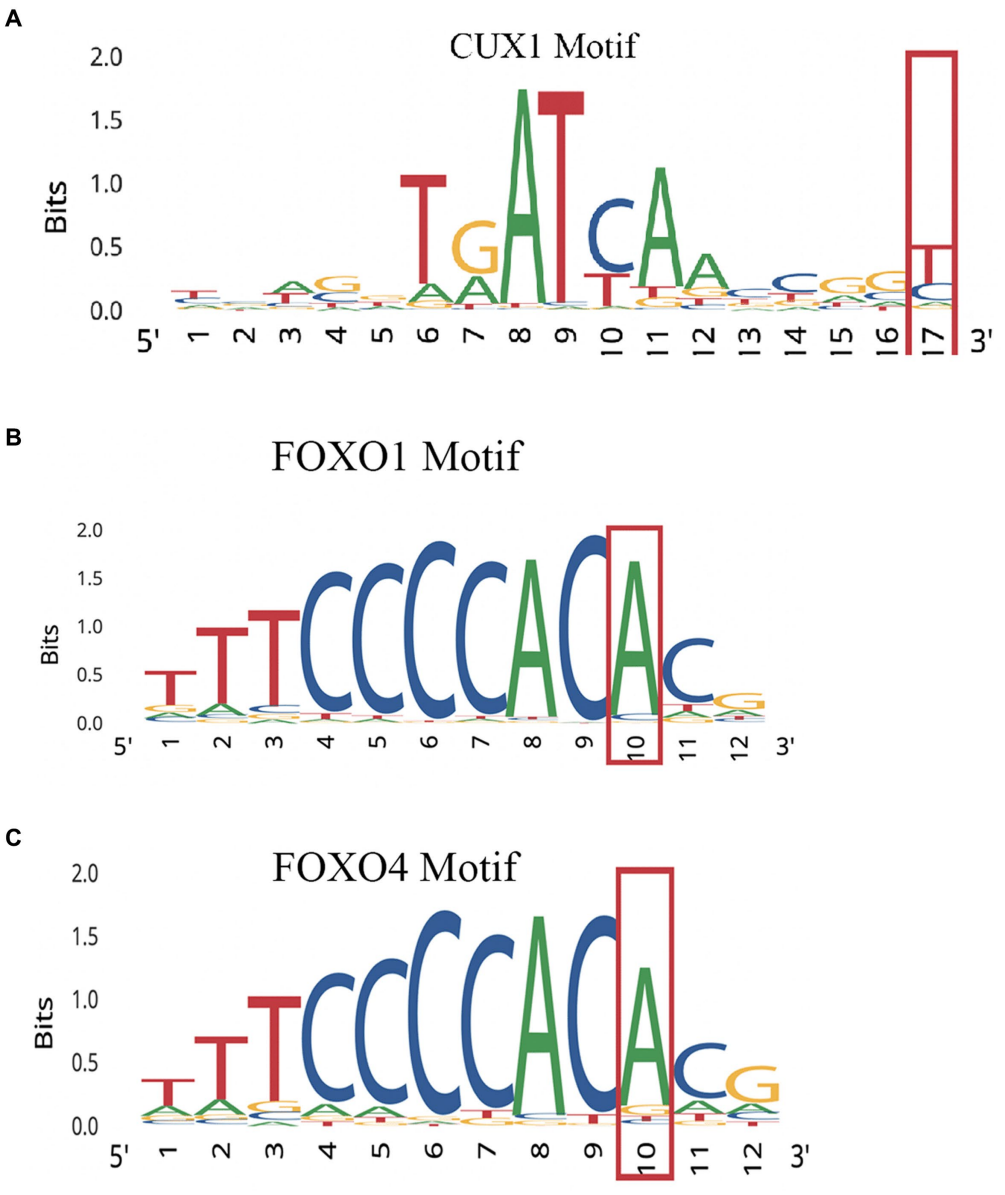
PWMs for upstream promoter variants showing affinity of regulatory protein. **(A)** NEK9 upstream variant rs12890371. **(B)** PPRX1 variant. **(C)** Variant of PRPF4B (rs11752006).

Performing variant analysis on upstream regions identified 66 variants in 61 genes. The distances of these variants varied from 1 bp to 916 bp upstream from the start codon. Parsing these further led us to identify variants in promoter regions of genes NIMA related kinase 9 (*NEK9*), paired related homeobox 1 (*PRRX1*), pre-mRNA processing factor 4B (*PRPF4B*), chymotrypsin like elastase 2A (*CELA2A*), RAB guanine nucleotide exchange factor 1 (*RABGEF1*) and cereblon (*CRBN*) that seemed to regulate some of the important genes involved in dopamine regulation. *NEK9* carried a variant (rs12890371) 56 bases upstream from the start codon, whereas *PRRX1*, *PRPF4B*, *CELA2A*, *RABGEF1,* and *CRBN* genes were found to harbor upstream variants at 194 bases (rs563991), 15 bases (rs11752006), 106 bases (rs5772642), 21 bases (rs1882655) and 29 bases (rs1672753) (Table 1) upstream. These variants had 1,000 genome (1000G) South Asian (SAS) frequencies of 0.5–1 and were all found to be homozygous in their occurrences. Further, these variants were integrated by leveraging the chromatin immuno-precipitation sequencing (ChIP-seq), quantitative trait loci (QTL), Formaldehyde-assisted isolation of regulatory elements (FAIRE-seq) and DNase I hypersensitive sites sequencing (DNase-seq), position weight matrix (PWM)/footprint and chromatin state experiment data points obtained from the ENCODE based regulome database (regulomedb) to identify the potential functional effects (Boyle et al., 2012). *NEK9* upstream variant, rs12890371 site was revealed to be bound by regulatory proteins of some of the immediate early response genes during substance use, such as JunD proto-oncogene (*JUND*), MYC proto-oncogene, bHLH transcription factor (*MYC*) and CREB binding protein (*CREBBP*). The variant of *PPRX1* was found to overlap a bivalent enhancer and a transcription start site (TSS), besides carrying a motif for cut like homeobox 1 (CUX1) transcription factor (TF) which is involved in controlling neuronal differentiation in the brain. It is also known to regulate dendrite development and branching, dendritic spine formation and control of synaptogenesis. Creating PWM revealed the variant to be located at 17th position with a bit-score of 0.5 (1.7 of 2.0 maximum) indicating moderate binding affinity with CUX1 at this location. Data from ChIP-seq revealed high affinity binding of enhancer of zeste 2 polycomb repressive complex 2 subunit (EZH2) TF to DNA motif overlapping this variant in bipolar neurons from male brains. Similarly, an upstream variant of PRPF4B (rs11752006) was found to overlap with forkhead box P2 (FOXP2) and GA binding protein transcription factor subunit alpha (GABPA) TF binding motifs in PFSK-1 and SK-N-SH neural cell lines. PWM revealed the variant location in the 10^th^ position with a higher binding affinity reaching 1.5 bit-score (1.5 of 1.8 maximum). This variant was under the regulatory binding region involving MYC, and YY1 transcription factor (YY1) (Figure 2). However, an upstream variant in *CELA2A* (rs5772642) showed relatively more of a quiescent chromatin state than a strong transcription state in substantia nigra, cingulate gyrus, anterior caudate, and dorsolateral prefrontal cortex besides containing a motif binding site for zinc finger protein 350 (ZNF350) with a very high binding affinity of 1.9 bit-score (1.7 of 2.0 maximum) (Supplementary Figure S1). In contrast, the upstream variant on *RABGEF1* (rs1882655) was found to overlap with an active TSS with relatively strong transcription. This variant site was found overlapping with FLII actin remodeling protein (FLII)/ Fli-1 proto-oncogene, ETS transcription factor (FLI1) and GA binding protein transcription factor subunit alpha (GABPA) TFs and comparisons with ChIP results revealed enrichment of GABPA and YY1 binding intersecting this variant site in SK-N-SH neural cell lines. Additionally, FAIRE-seq and DNase-seq analysis revealed the open accessibility at regulatory region chr7:66146587–66,148,147 overlapping the rs1882655 variant location in several cell types from cerebellum and frontal cortex, besides other organs. The Footprinting information revealed the variant rs1882655 to also overlap with FLI1, and GABPA binding regions (Pique-Regi et al., 2011; Boyle et al., 2012). The rs1672753 variant present upstream of *CBRN* is predominantly bound by transcription regulators, CCCTC-binding factor (CTCF) (normal human astrocytes and middle frontal area 46), SWI/SNF related, matrix associated, actin dependent regulator of chromatin, subfamily a, member 4 (SMARCA4) (biopolar spindle neuron), YY1 (SK-N-SH neural cell lines), GATA binding protein 2 (GATA2) (SK-N-SH neural cell lines) and GABPA (SK-N-SH neural cell lines). This variant influences the chromatin to have an active TSS and seems to overlap with enhancer and repressor protein binding sites. The PWM revealed mild affinity with the E2F1 protein binding motif (Supplementary Figure S1).

**Table 1:** List of upstream promoter variants identified in the current study of SUDs.

Chr	Start	End	Ref	Alt	Variant location	Gene	Distance from start codon	1000G_SAS	Zygosity	rsID	Experimental evidence
chr14	75,593,839	75,593,839	T	C	Upstream	NEK9	56	0.47	hom	rs12890371	Likely to affect binding and linked to expression of a gene target
chr1	170,633,119	170,633,119	T	G	Upstream	PRRX1	194	1	hom	rs563991	Minimal binding evidence
chr6	4,021,554	4,021,554	A	G	Upstream	PRPF4B	15	1	hom	rs11752006	Likely to affect binding
chr1	15,783,117	15,783,117	-	A	Upstream	CELA2A	106	0.99	hom	rs5772642	Less likely to affect binding
chr7	66,147,057	66,147,057	T	C	Upstream	RABGEF1	21	0.8	hom	rs1882655	Likely to affect binding
chr3	3,221,430	3,221,430	C	T	Upstream	CRBN	29	0.79	hom	rs1672753	Likely to affect binding

#### 3.1.2 CH variant analysis

The availability of family-and pedigree-based variant information led us to perform CH analysis to identify heterogeneous recessive alleles that can cause SUDs in a heterozygous state. The PhenoDB program was used to estimate the compound heterozygous alleles with a MAF of 0.01 for exonic variants (nonsynonymous, stopgain, stoploss, splicing and frameshift indels). Families 1, 3, 4 and 5 were found to carry 13, 2, 9, and 14 variants with this filtering, while family 2 did not show any CH variants. Among these, screening for SUDs-relevant variants narrowed to 6 variants in 3 genes in 2 families (1 and 5). These genes were identified by juxtaposing them in the neuropeptide-neurotransmitter network axis. Family 1 showed 4 frameshift deletions in 2 genes, *LNPEP* and *LRP1*, while family 5 showed 2 frameshift deletions in the *TBX2* gene (Supplementary Table S2). All the deletion variants were heterozygous and appeared to be in trans condition.

**Figure 3:**
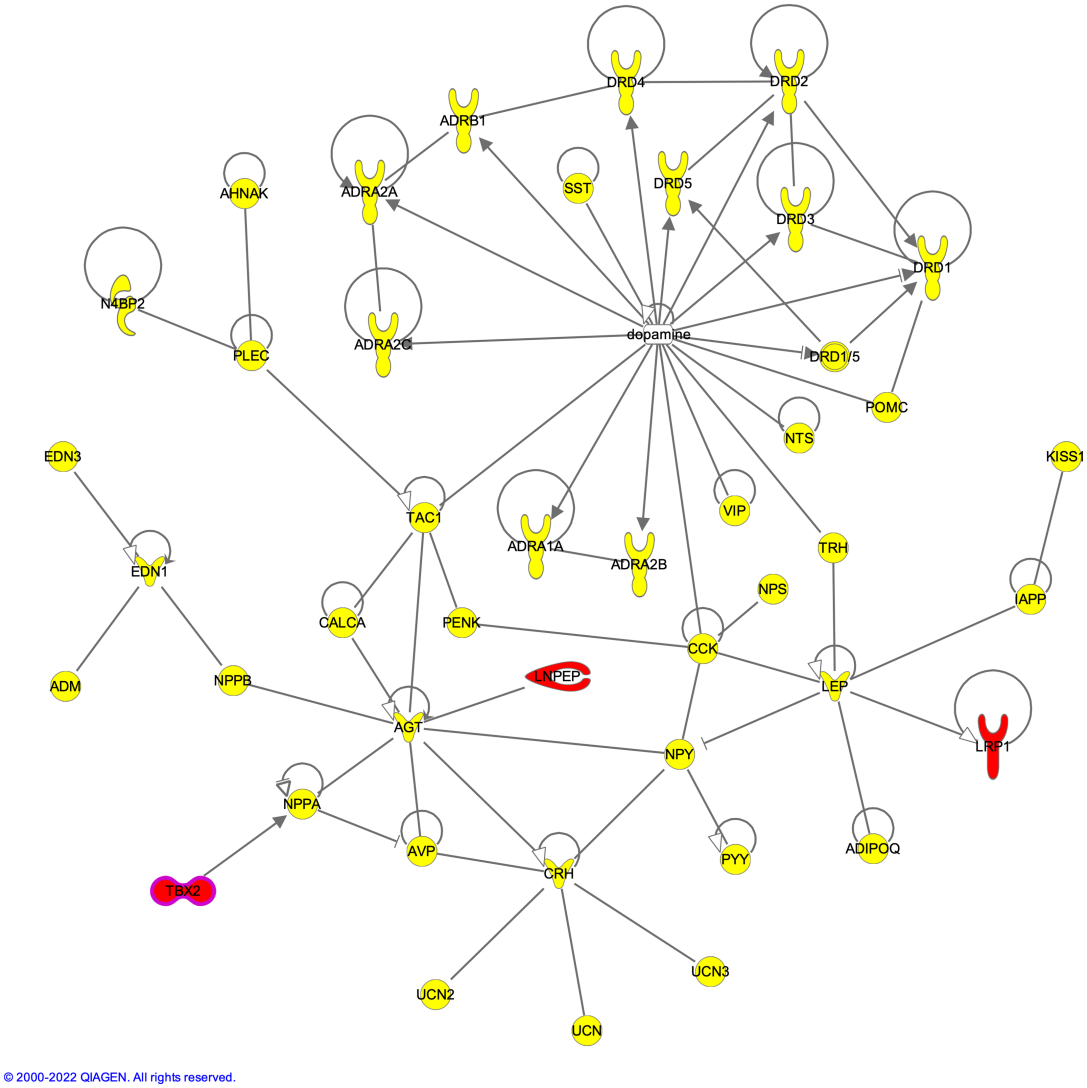
Compound heterozygous genes LNPEP, TBX2 and LRP1 in the dopaminergic axis of neuropeptide-neurotransmitter network.

*LNPEP* carried 2 frameshift deletions, the first deletion was in exon 2 of isoforms NM_005575 (c.428delA:p.K143fs) and NM_175920 (c.386delA:p.K129fs), while the second deletion (also in exon 2) caused the frameshift at 143^rd^ aa position in isoform NM_005575 (c.427_428del:p.K143fs) and at 129^th^ aa position NM_175920 (c.385_386del:p.K129fs). These deletions caused the amino acid (aa) frame to shift, creating a stop codon 34 codons downstream at the 149th aa position in isoform NM_005575 and 163^rd^ aa position in isoform NM_175920 (Supplementary Table S2). Similarly, *LRP1* also showed 2 frameshift deletions in exon 62 of isoform NM_002332 (c.9928delG:p.G3310fs; c.9927_9928del:p.P3309fs) causing a frameshift and disrupting the splicing site at the end of exon 62. Correspondingly, frameshift deletions in *TBX2* were found in exon 7 in isoform NM_005994 (c.2005_2006del:p.A669fs and c.2006delC:p.A669fs) (Supplementary Table S2). While the single bp deletion of G caused a stop codon at the 696^th^ aa position, the 2-bp deletion of GC caused the stop codon at the 686th position. Network analysis placed these SUD-relevant genes in the direct proximity of the neurotransmitter and neuropeptide axis; LNPEP was found to indirectly interact with angiotensinogen (AGT) through the enzymatic pathway that leads to the production of angiotensin IV, a direct ligand of LNPEP; LRP1 with leptin (LEP); and TBX2 with natriuretic peptide A (NPPA) genes (Figure 3).

#### 3.1.3 Homozygosity mapping

**Figure 4:**
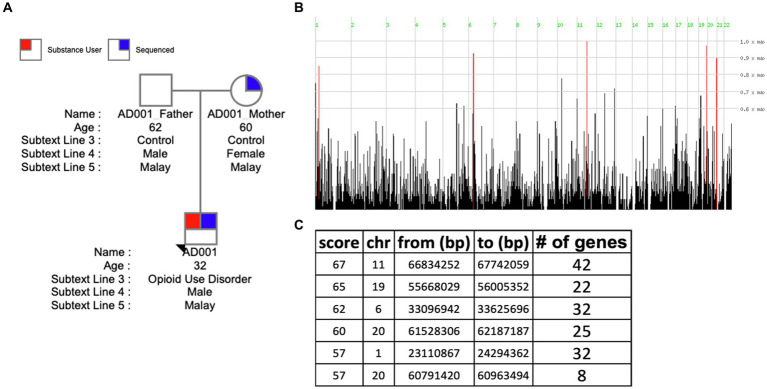
Homozygosity mapping in family 1. **(A)** Pedigree of Family 1 (AD001). **(B)** Visualizing the distribution of homozygous regions in the genome of AD001 proband. **(C)** Table listing the identified homozygous regions.

Homozygosity mapping was performed on probands and their family member cohorts and case–control (all probands vs. related members) cohort. Table 2 details the homozygosity regions identified in families and genes annotated in those regions. HM revealed the presence of stretches of homozygosity across all families. Family 1 showed 6 homozygous stretches of 161 genes (Figure 4). Similarly, Supplementary Figures S2, S3 show homozygosity stretches for families 5 and 3 consisting of 7 (187 genes) and 8 (248 genes) blocks, respectively. Families 2 (74 genes) (Supplementary Figure S4) and 4 (62 genes) (Supplementary Figure S5), however, showed only a few regions. Screening for SUDs-related genes (selection criteria is provided in methods) led us to identify 6 genes: *CHRNA4* (chr 20) and *CNR2* (chr 1) in family 1 with a homozygous score of 60 and 32, respectively; *GABBR1* (chr 6) and *NPAS4* (chr 11) in family 3 with a homozygous score of 80; while genes *DRD4* and *SCT* were identified in family 5 both in chromosome 11 with a score of 70 (Table 2). Furthermore, clustering these families into a case–control cohort and performing the HM revealed 47 homozygous haplotype blocks in chromosomes 2, 22, 3, 16, 15, 12, and 17 (Supplementary Figure S6). Supplementary Table S3 provides the HM specifics regarding the runs of homozygosity, region-and gene-based annotations, and HM scores along with the list of genes.

**Table 2:** Homozygosity blocks identified through homozygosity mapping in proband and related family cohort as well as case–control (all probands vs. related/unrelated family members) cohorts.

Family ID	Number of homozygous blocks	Number of genes in them	SUDs genes in homozygous blocks	Chromosomes that carry SUDs genes
1	6	161	CHRNA4, CNR2	20, 1
2	1	74	–	–
3	8	248	GABBR1, NPAS4	6, 11
4	2	62	–	–
5	7	187	DRD4	11
Case–Control	47	47	–	–

##### 3.1.3.1 Overlap with SUDs GWAS Associations

Several single nucleotide variants previously associated with SUDs were identified in the probands. Supplementary Table S4 details these genotypes along with the associated traits. A total of 20 SNPs were found in the cohort that was reported to be associated with GWAS of SUDs. SNP, rs324420 (C;A), associated with imparting susceptibility to polysubstance use was found in all 5 probands, while SNP, rs1229984 (T;C), associated with alcohol consumption was found in 4 of the 5 probands. This cohort showed the presence of 3 SNPs each for susceptibility toward alcohol addiction/consumption, and nicotine addiction, while 1 SNP each for opioid dependence and polysubstance abuse (Supplementary Table S4). These variants were found in genes, fatty acid amide hydrolase (*FAAH*), transferrin (*TF*), *ADH1B*, *OPRM1* and opioid growth factor receptor (*OGFR*). These variants showed varying rate of penetrance in related and unrelated family members.

##### 3.1.3.2 miRNA variant analysis

Annotating the variants in microRNA (miRNA) genes identified 3 variants in the coding regions (Supplementary Table S5). *mir-548 U* was found with G to A transition (rs2894842) at 57254955 bp position, while *mir-532* showed 2 variants both A to G transitions at 49767832 bp (rs456615) and 49,767,835 bp (rs456617) positions (Supplementary Table S5). Among other miRNA variants, these three were of particular interest due to their involvement in positive regulation by immediate early response genes, *MYC* and Jun proto-oncogene, AP-1 transcription factor subunit (*JUN*). The variant rs2894842 was found to be heterozygous with a 1000G South Asian (SAS) frequency of 0.5, while variants, rs456615 and rs456617 were homozygous with a frequency of 1 (Supplementary Table S5).

### 3.2 Genotyping variants concordant in all probands

In this second approach, the variants from all probands were genotyped and further filtered to include only those that were concordant amongst all probands (Figure 1). The resulting set of variants were merged and annotated without any Minor allele frequency (MAF) filtering. This approach led us to identify only two variants that were enriched in all SUDs probands (Table 3). A frameshift insertion of G in the *GRIA3* gene was found to cause the frame to shift at 129^th^ amino acid (aa) position leading to a truncated GRIA3 protein, 17 aa downstream to the mutated site. This truncation mutation was found in a homozygous state with a frequency of 1 in South Asians (SAS) of 1000G (1,000 genome) project. Furthermore, a G to A nonsynonymous substitution was observed in the *NCOR1* gene at 16068396 bp position resulting in a substitution of serine with leucine with a 1000G SAS frequency of 0.48 (Table 3).

**Table 3:** Significant protein-function altering variants identified in SUDs probands.

Chr	Start	End	Ref	Alt	Gene	Variant type	AA change	1000G_SAS (%)	Zygosity
chrX	122,336,600	122,336,600	-	G	GRIA3	Frameshift insertion	NM_001256743:exon3:c.382dupG:p.P129Afs*17	1	Hemizygous
chr17	16,068,396	16,068,396	G	A	NCOR1	Non-synonymous SNV	NM_001190438:exon2:c.C188T:p.S63L NM_001190440:exon4:c.C515T:p.S172L NM_006311:exon5:c.C515T:p.S172L	0.48	Heterozygous
chr19	51,170,706	51,170,706	A	G	SHANK1	Non-synonymous SNV	NM_016148:exon22:c.T4511C:p.V1504A	0.59	Heterozygous

### 3.3 Trio exome analysis of *de novo* variants

Family-based variant filtering and genotyping was performed using the third approach (Figure 1). Here, variants in each proband were compared (by position) against variants of corresponding family members resulting in only the unique variants for the probands. These VCFs containing unique variants of all probands were later merged into a multi-sample VCF file to identify concordant variants. This filtering approach led us to no variants at the end of the analysis; therefore, we performed variant intersection on pairwise, trio-, quartet-, and quintet comparisons between the probands to build a matrix to identify the highest variant concordance among the cohort. Based on the number of shared variants (by position) among probands led us to create a matrix identifying *n* = 3 (subjects AD001, AD002 and AD005) probands that shared a higher number of shared variants among themselves (Figure 1). Using this filtering metric, we repeated genotyping in *n* = 3 probands for subjects AD001, AD002, and AD005. This led us to identify two heterozygous nonsynonymous variants in two genes, hornerin (*HRNR*) and *SHANK1* with a MAF of 0.12 and 0.59, respectively. *SHANK1* showed an A to G change at 51170706 bp resulting in a valine to arginine substitution at 1504 amino acid (aa) position (Table 3).

Performing haploinsufficiency analysis on the genes bearing heterozygous variants from this study showed 90% of them to be dosage sensitive. Genes *NCOR1, ADH1B*, *SHANK1*, *TF*, *OPRM1*, and *GABBR2* are highly dosage sensitive, while huntingtin interacting protein 1 (*HIP1*) and *OGFR* were moderately haploinsufficient (Table 4).

**Table 4:** List of variants identified in SUDs relevant genes along with the method used, known/novel status and haploinsufficiency evidence.

Method	Genes	Zygosity	Known/Novel	DECIPHER evidence
Genes imparting susceptibility to substance use				
Protein-function altering variants	GRIA3	Homozygous	Known	Not Applicable
	NCOR1	Heterozygous	Known	Haploinsufficient
	SHANK1	Heterozygous	Novel	Haploinsufficient
ClinVar variant genotyping	FAAH	Heterozygous	Known	Not haploinsufficient
	TF	Heterozygous	Known	Haploinsufficient
	ADH1B	Heterozygous and Homozygous	Known	Heterozygous are not haploinsufficient
	OPRM1	Heterozygous	Known	Haploinsufficient
	ADH1C	Homozygous	Known	Unknown
	HIP1	Heterozygous	Known	Moderately haploinsufficient
	CHRNA4	Heterozygous	Known	Not haploinsufficient
	GABBR2	Heterozygous	Known	haploinsufficient
	OGFR	Heterozygous	Novel	Moderately haploinsufficient
Variants in genes influencing SUDs traits				
Homozygosity mapping	CHRNA4	Homozygous	Known	Not applicable
	CNR2	Homozygous	Known	Not applicable
	GABBR1	Homozygous	Known	Not applicable
	NPAS4	Homozygous	Known	Not applicable
	DRD4	Homozygous	Known	Not applicable
Upstream promoter analysis	NEK9	Homozygous	Novel	Not applicable
	PRRX1	Homozygous	Novel	Not applicable
	PRPF4B	Homozygous	Novel	Not applicable
	CELA2A	Homozygous	Novel	Not applicable
	RABGEF1	Homozygous	Novel	Not applicable
	CRBN	Homozygous	Novel	Not applicable
miRNA variant analysis	mir548U	Heterozygous	Novel	Unknown
	mir532	Homozygous	Novel	Not applicable
Compound heterozygosity analysis	LNPEP	Homozygous	Novel	Not applicable
	LRP1	Homozygous	Novel	Not applicable
	TBX2	Homozygous	Novel	Not applicable

## 4 Discussion

Performing genetic and bioinformatic analysis on WES datasets from 5 family trios with SUDs identified a spectrum of variants in genes. These variants were identified by integrating a multitude of approaches, including traditional variant filtering followed by compound heterozygosity analysis, homozygosity mapping, upstream promoter variant analysis, miRNA variant analysis, genotyping of the known SUD variants, and lastly, haploinsufficiency analysis. Among the several variants identified in a total of 27 genes for SUDs phenotype in this study, 20 variants had been previously reported in the ClinVar database for SUDs. A substantial number of genes from this study were found implicated in SUDs, such as *CHRNA4*, *CNR2*, *GABBR1*, *DRD1*, *NPAS4*, *ADH1B*, *ADH1C*, *OPRM1* among others, while the study also identifying several novel genes. While the findings of our study are promising, one limitation is the relatively small sample size of only 15 subjects, which may not be representative of the larger population. However, it is important to note that the cohort is from a pilot study performed to establish the feasibility of using high-resolution whole exome sequencing methodology to identify candidate genes for SUDs. The use of this methodology has allowed us to identify genetic variants with high accuracy and resolution.

### 4.1 Variants in receptor genes of excitatory synaptic transmission

Traditional variant filtering, followed by compound heterozygosity analysis identified high-confidence protein-altering variants in genes, such as *GRIA3*, *NCOR1* and *SHANK1*. *GRIA3* is a receptor for glutamate neurotransmitter. This receptor plays an important role in excitatory synaptic transmission. Several studies have convincingly implicated genetic variants of *GRIA3* in polysubstance use. Iamjan et al. (2018) showed variants in GRIA3 to be associated with methamphetamine dependence and methamphetamine-induced psychoses. Lo et al. (2016) performed a genome-wide scan in a breed of mice with heightened alcohol seeking behavior, regarded as an alcohol preference trait. Their findings revealed the presence of variants in Gria3 confirming their involvement in the efficiency of excitatory communication and synaptic memory in alcohol preference and dependence. Furthermore, Davies et al. (2017) reported mutations in the *GRIA3* gene in a family with severe sleep and circadian rhythm disruption. This and several other evidences support the role of circadian rhythm in modulating reward processing, demonstrating the direct role of circadian systems in substance use (Hasler et al., 2012). Animal studies involving the Gria3 knock-out mice have shown alterations in exploratory behavior (Sanchis-Segura et al., 2006), increased social and aggressive behavior (Adamczyk et al., 2012), and certain forms of motor learning deficits (Gutierrez-Castellanos et al., 2017). These layers of evidence suggest a link between changes in GRIA3 and substance use behaviors. The identification of a frameshift mutation in GRIA3 among the individuals from the five family trios further indicates its potential influence on susceptibility to and the sustenance of substance use. This approach underlines the need for further research to fully understand the gene’s role in these complex behaviors.

Another lesser-known gene, *NCOR1* was found with a non-synonymous variant spanning the coiled-coil domain across all 5 probands. *NCOR1* has been found to express across brain regions, including the GABAergic neurons (Zhou et al., 2019). Pathogenic mutations in *NCOR1* have been reported in a range of neuropsychiatric domains such as poor motor coordination, aggressive attitude, moderate learning difficulties among several others (Firth et al., 2009; Zhou et al., 2019). Zhou et al. (2019) further performed animal studies involving mice with DAD (domain of NCOR1) knock-in mutations that showed significant memory deficits along with reduced, social interactions and anxiety levels. Depletion of NCOR1/2 specifically in GABAergic neurons was found to reinforce the memory deficits combined with reduced gamma-aminobutyric acid type A receptor subunit alpha2 (GABRA2) expression. Functionally, NCOR1 recruits histone deacetylase 3 (HDAC3) resulting in the repression of basic helix–loop–helix ARNT like 1 (BMAL1) expression affecting circadian rhythms (Saad et al., 2021). Mutations in the DAD domain of NCOR1 have been shown to impede the binding and activation of HDAC3 causing dysregulation of clock genes and circadian behavior (Alenghat et al., 2008). Since altered circadian behavior is known to be a significant risk contributor toward the development of SUDs, qualifying NCOR1 as an excellent candidate for further investigations in the context of SUDs. Further, NCOR1 also exhibits ligand-dependent interaction with vitamin D receptor (VDR) (Tamura et al., 2017); our pathway analysis further revealed VDR to regulate EDN1, an important regulator of immediate early response genes of substance use such as, MYC, FOSL1 and FOSB, in addition to regulating key neuropeptides, adrenomedullin (ADM), and natriuretic peptide B (NPPB). The downstream relationship of NCOR1 to known SUDs influencing dopaminergic activators suggests a potential role in increasing susceptibility to substance use, necessitating further research.

**Figure 5:**
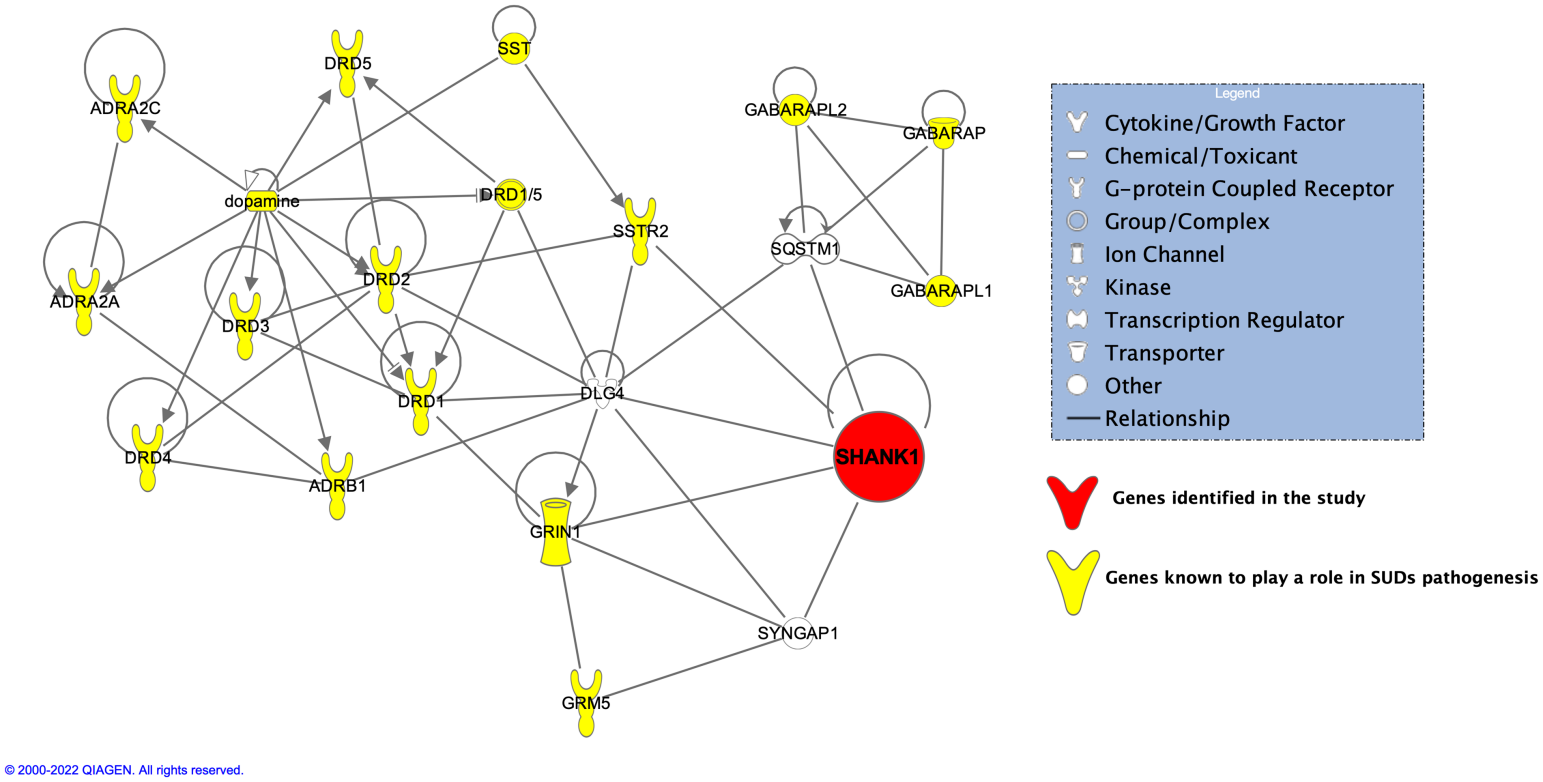
IPA on SHANK1 and its neighbors put SHANK1 directly on the axis of dopaminergic and glutamatergic pathways.

In addition to *GRIA3*, and *NCOR1*, another interesting gene, *SHANK1* was found present exclusively in 3 of the probands (Probands 1, 2, and 5) but none in the related or unrelated family controls. *SHANK*1, known as SH3 And Multiple Ankyrin Repeat Domains 1 is known to be exclusively expressed in the brain belonging to the Shank family of postsynaptic scaffold proteins found in abundance in the postsynaptic regions of excitatory synapses. Shank promotes the maturation and enlargement of dendritic spines. Pal and Das (2013) performed animal studies on Shank1 showing its involvement at both transcript and protein levels in regulating spine morphology induced by chronic morphine exposure resulting in addiction. Results from amphetamine (AMPH) and 3,4-methylenedioxymethamphetamine (MDMA) substance treated Shank1^−/−^ by Sungur et al. (2018) showed the mice display reduced psychostimulant-induced hyperactivity compared to controls. Performing IPA on SHANK1 and its neighbors put SHANK1 directly on the axis of dopaminergic and glutamatergic pathways (Figure 5). SHANK1 was found to have crosstalk between members belonging to glutamatergic pathways [glutamate ionotropic receptor NMDA type subunit 1 (GRIN1)] (Naisbitt et al., 1999), postsynaptic proteins (DLG4, SYNGAP1) (Arbuckle et al., 2010; Li et al., 2016, 2017; Wilkinson et al., 2017), the regulator of activation of the nuclear factor kappa-B (NF-kB) signaling pathway (SQSTM1/p62) (Rezvani et al., 2007), and lastly, with the receptor of a neuropeptide, somatostatin (SSTR2) (Kreienkamp et al., 2000; Pagel et al., 2005). SSTR2 stimulates neuronal migration, axon outgrowth and participates in neurotransmission and secretion (Zitzer et al., 1999). Deliberating on these evidences, SHANK1 may influence susceptibility to substance use by affecting neurotransmission pathways. While the specific pathogenic impact of the SHANK1 variant remains uncertain, its exclusive presence in probands suggests a potential role in contributing to functional abnormalities that, along with other variants, might affect susceptibility to substance use.

### 4.2 Promoter variants in genes regulating immediate early response and neurotransmitter-neuropeptide axis

Supplementary Figure S7 shows the IPA performed on upstream promoter variants identified in genes, *NEK9*, *PRRX1*, *PRPF4B*, *CELA2A*, *RABGEF1* and *CRBN*. These genes are relatively unknown in the context of SUDs but showed their direct involvement by regulating genes involved in immediate early response and neurotransmitter-neuropeptide axis. *NEK9* showed a promoter variant that was 56 bp upstream of the open reading frame (ORF) and has been shown to likely affect the binding affinity of transcription regulators. NEK9 is a kinase signal transducer from the GABA cluster consisting of GABA type A receptor-associated protein (GABARAP), GABA type A receptor associated protein like 1 (GABARAPL1) and GABA type A receptor associated protein like 2 (GABARAPL2) cell membrane proteins (Behrends et al., 2010; Shrestha et al., 2020). Dysfunctional expression of GABA_A_Rs has been correlated with substance use (Ye et al., 2021). Since phosphorylation of γ2-GABA_A_R is known to result in differential modulation of GABA_A_ receptors binding to GABARAP and AP2, it suggests that altered dosages of NEK9 may hinder the regulation of synaptic localization of GABA_A_Rs (Ye et al., 2021). The upstream promoter variant of PRRX1 was in the CUX1 motif binding site 194 bp upstream from the start ORF. IPA provided evidence of clusters of adrenergic receptors, ADRA1A, ADRA1D and ADRA1B, to regulate the expression of PRRX1 mRNA (Gonzalez-Cabrera et al., 2003). PRRX1 is a transcriptional regulator known to regulate FOS, one of the immediate early response genes. A protein–protein complex consisting of general transcription factor IIi (GTF2I) and PRRX1 binds to the serum response element (SRE) in the upstream promoter region of the FOS gene leading to increased transcription (Grueneberg et al., 1997).

Similarly, variant rs11752006 was found 15 bp upstream of *PRPF4B* ORF site. This variant showed a high affinity binding site for motifs, FOXO1 and FOXO4. Interestingly, PRPF4B cross-talks with NCOR1 (Dellaire et al., 2002) which is one of the other genes discovered in the current study and described above, to have protein-function altering variants. Variant rs5772642 identified 106 bp upstream from CELA2A with a higher binding affinity site for ZNF350. CELA2A has been shown to be under the transcriptional regulation of yet another immediate early response gene, MYB (Ramsay and Gonda, 2008). In turn, MYB has been found to bind to demethylated sites of BDNF and is involved in cocaine-triggered seeking behavior (Tian et al., 2016). RABGEF1 showed an expression altering variant just 21 bp upstream from the ORF overlapping the GABPA and FLII/II motifs. RABGEF1 protein functions in endocytic membrane fusion and membrane trafficking, while endosomes in presynapse enable the vesicles to bud off the endosome forming neurotransmitter vesicles. Deliberating on these two pieces of information, RABGEF1 protein seems to be involved in the endocytosis of trafficking and processing of neurotransmitter release. IPA further provided evidence toward the direct and indirect crosstalk of RABGEF1 with a broad array of adrenergic (ADRB2), GABAergic (GABRA1, GABRA2, and GABBR2), dopaminergic (DRD1 and DRD2), and glutamatergic (GRM1, GRM2, and GRM3) receptors. Interestingly, RABGEF1 interacts with HTT, which was identified in one of our previous studies to be having most downstream connections with known SUDs genes than any other in the network, while OPRM1 is its upstream regulator (Veerappa et al., 2021). These layers of evidence reflect on the roles of *RABGEF1* in SUDs etiology. Lastly, the cereblon (CRBN) gene was found carrying a variant rs1672753, 29 bp upstream overlapping with E2F1 motifs and likely to affect the binding of regulatory proteins. Cereblon is involved in maintaining glutamate release at presynapses, subsequently altering memory and learning. Cereblon has been speculated to be involved in regulating anxiety-like behaviors (Rajadhyaksha et al., 2012), and since anxiety and substance use co-occur frequently (Smith and Book, 2008), the role of cereblon in inducing anxiety prompted substance use needs further investigation. In addition, cereblon, a crucial immediate early response gene detected in substance use is shown to have crosstalk with several transcription regulators, sequestosome 1 (SQSTM1), growth factor receptor bound protein 2 (GRB2) and JUN. It can be hypothesized that promoter variants of NEK9, PRRX1, PRPF4B, CELA2A, RABGEF1, and CRBN might influence the expression of downstream genes, potentially affecting the synaptic localization of GABA_A_Rs, increasing FOS gene transcription, and altering neurotransmitter release, which could play a role during the pre-initiation phases of substance use, or throughout substance use, and/or during withdrawal periods. This suggests a nuanced role for these variants in the dynamics of SUDs, underscoring the necessity for additional experimental work to validate their precise impact on the pathophysiology of SUDs.

### 4.3 Accumulated effects of multiple recessive genes contributing toward SUDs

Performing homozygosity mapping identified stretches of homozygous haplotypes enriched with recessive variants. Among several homozygous haplotypes detected, Table 2 describes the SUDs-related regions and genes within them. Studies on SUDs involving humans and other animals have shown receptors, *GABBR1* in modulating synaptic GABA (Enoch et al., 2016); *CNR2* in the reward system (Ishiguro et al., 2007); *CHRNA4* in establishing dependence, withdrawal, and affective symptoms (Lazary et al., 2014); *DRD4* in predisposing toward severe dependency (Lusher et al., 2001) and transcription regulator, neuronal PAS domain protein 4 (*NPAS4*) in playing a role in reward-relevant learning and memory processes (Taniguchi et al., 2017). The genes identified within the homozygous regions of the probands, could play a significant role in influencing a broad spectrum of SUDs phenotypes. Further research is required to validate this hypothesis.

Variants in non-protein coding RNA genes led us to emphasize our attention toward the heterozygous variant in *mir-548 U* (rs2894842) and homozygous variants in *mir-532* (rs456615/rs456617). Interestingly, the crucial immediate early response genes, *JUN* and *MYC*, are known to positively regulate the expression of *miR-548 U* (mir-570) and *mir-532* (mir-188), respectively (Marzi et al., 2012; Baker et al., 2019). Though these miRNAs are known to be under the regulation of JUN and MYC, this is the first study to show the involvement of these two miRNAs in the background of SUDs (Supplementary Figure S8). These influences and regulations are further compounded by the presence of compound heterozygous frameshift deletions in *TBX2*, *LNPEP*, and *LRP1*. TBX2 binds the promoter fragment (−273--236) containing an Nkx-2.5 response element (NKE) and a T-box binding site in the NPPA gene and decreases its transcription (Habets et al., 2002), LNPEP (IRAP) was found to indirectly interact with angiotensinogen (Albiston et al., 2001), and LRP1 regulates crucial leptin signaling gene, *LEP* (Liu et al., 2011). The brain renin angiotensinogen system (RAS) regulates endocrine, autonomic, and behavioral responses to stress in the cortical and limbic systems (Mcewen, 2007; Sommer and Saavedra, 2008). Several experimental evidences exist to support the role of LNPEP-AGT axis in alcohol dependence. Upregulation of *AGT* has been reported from different rodent breeds for high ethanol preference (Kiianmaa et al., 1991; Saba et al., 2006; Sommer et al., 2006). Animal studies involving genetic modification of the RAS have shown angiotensinogen to positively modulate spontaneous ethanol consumption (Maul et al., 2001, 2005). LNPEP is a zinc-dependent aminopeptidase that cleaves, inactivates neuropeptides including oxytocin, and catalyzes the conversion of angiotensinogen (Mizutani et al., 1982; Yokosawa et al., 1983; Albiston et al., 2001; Rioli et al., 2003). This regulator of several neuropeptides is anticipated to be non-functional due to compound heterozygous mutations disrupting the extracellular domain from one allele, and the other allele disrupting the helical and signal-anchor for type II membrane structure. Further, studies have shown inhibition, knockdown and deletion of leptin signaling by targeting leptin receptors led to an increase in dopamine levels, anxiety levels and enhanced the cocaine-conditioned reward. LRP1 bound by leptin-inducing leptin signaling was found bearing frameshift deletions truncating the protein. Though we cannot assess the pathogenic contribution of this mutant allele, we can speculate its influence on SUDs based on its close association with leptin signaling.

Genotyping for variants reported in earlier SUDs studies led us to identify some of the crucial variants in genes, *FAAH*, *TF*, *ADH1B*, *OPRM1*, *ADH1C*, *HIP1*, *CHRNA4*, *OGFR,* and *GABBR2* (Supplementary Table S4). These genes have been convincingly associated with SUDs in attributing polysubstance use risk; however, no functional assessments exist to determine the pathogenic effects of these variants. Studies have assumed a certain degree of risk for substance use in chronic users with these alleles, but it requires further functional validation to understand the definitive risk.

**Figure 6:**
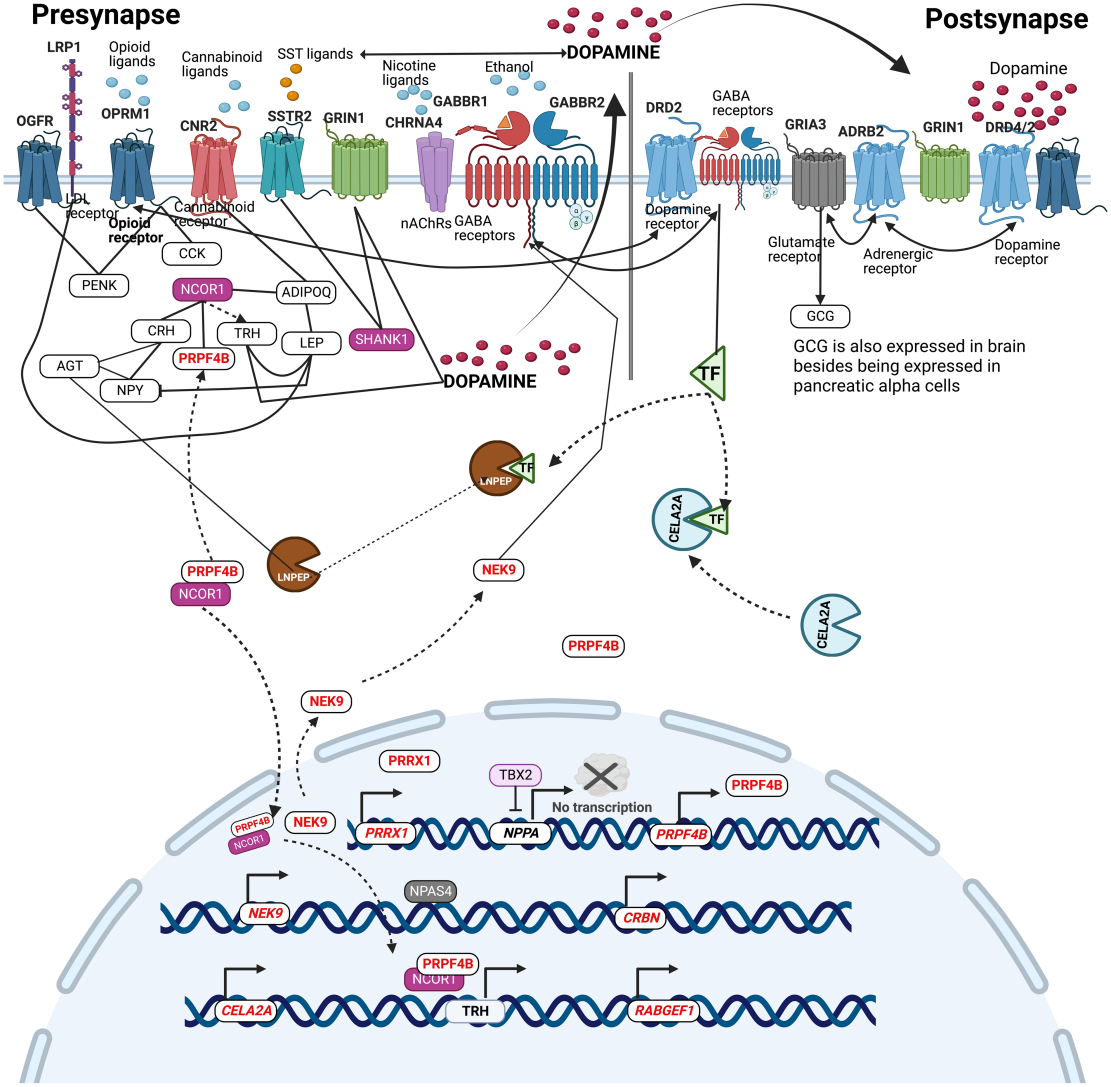
Integrative pathway analysis displays the existence of collaborative relationships between the genes carrying variants in SUDs patients from the current study. The pathway describes the molecular location, and the molecular relationships that exists among SUDs genes with variants in the context of pre-and post-synapses.

An attempt was made to integrate all the SUDs associated genes bearing variants identified in the current study to understand their molecular crosstalk. Figure 6 describes the molecular location, and the molecular relationships that exist among SUDs genes with variants in the context of pre-and post-synapses. Receptor genes, *GRIA3*, *OPRM1*, *CHRNA4*, *GABBR2,* and *OGFR*, were identified to carry variants in the current study. These receptors bind with glutamate neurotransmitter, opioids, nicotine, ethanol, and opioids growth factor, respectively. Dopamine binding to DRD2/4, modulates ADRB2, which in turn regulates the GRIA3 activity in the production of glucagon (GCG). Glucagon works besides insulin to control blood sugar levels. In the context of this study, insulin acts as a reporter of internal environments in the modulation of reward (Daws et al., 2011). Ethanol binds to GABBR2, causing receptor clustering with DRD2 mediated by TF (Kumar et al., 2004; Jung and Harris, 2006; Borghese et al., 2016). TF is processed enzymatically by CELA2A and LNPEP. Similarly, the activity of LNPEP is indirectly regulated through the enzymatic conversion of AGT into angiotensin (Albiston et al., 2001). In this way, the proximity of CELA2A and LNPEP with genes belonging to neurotransmitter-neuropeptide axis of the dopaminergic circuit signifies their participation in SUDs etiology. Chronic opioid exposure in the rat brain and spinal cord has shown to increase cholecystokinin (CCK) mRNA and CCK immunoactive peptide in the regions of hypothalamus and the spinal cord (Ding and Bayer, 1993), while Cadet et al. (2016) found that methamphetamine administration in rats led to increased expression of proenkephalin mRNA (PENK) in the Nucleus Accumbens indicating that exposure to drugs can modulate the expression of these neuropeptides in brain regions. NCOR1 regulates a plethora of neuropeptides and non-neuropeptides such as PRPF4B. *PRPF4B*, *PRRX1*, *NEK9*, *CELA2A*, *RABGEF1*, and *CRBN* genes were found to have upstream promoter variants (Table 1). NCOR1 and PRPF4B protein complex binds to the *TRH* gene’s upstream promoter regions, enabling its regulation. *LNPEP*, *LRP1* and *TBX2* were found to carry CH variants. TBX2 is known to negatively regulate NPPA neuropeptide, however, the compound heterozygosity variant alleles of *TBX2* may cause reduced inhibition, leading to dysregulated activations of NPPA (Figure 6). This integrative pathway displays the existence of collaborative relationships among the genes carrying variants in SUDs patients from the current study.

In conclusion, we identified several SNPs and rare protein-function altering variants that play a major role in the axis of dopaminergic circuits (Figure 6). While deleterious mutations in some of these genes result in manifestation of neurodevelopmental phenotypes, other benign variants in the same genes may result in subtle functional consequences that perhaps influence the protein activity, and function accumulating towards cumulative effect on substance use traits. These variants were also found to be overrepresented among genes participating in the neurotransmitter-neuropeptide axis, suggesting pleiotropic influences in the development and sustenance of chronic substance use (Figure 6). We also note a greater frequency of variants in genes involved in the structural and functional integrity of synapse receptors. This study demonstrates the presence of a diverse set of haploinsufficient variants in varying frequencies, demonstrating the presence of extraordinary collaboration that exists among them in attributing risk and modulating severity to SUDs.

## Data availability statement

The original contributions presented in the study are included in the article/Supplementary material, further inquiries can be directed to the corresponding author.

## Ethics statement

The studies involving humans were approved by the Domain Specific Review Board (DSRB Ref: 2016/01111). The studies were conducted in accordance with the local legislation and institutional requirements. Written informed consent for participation was not required from the participants or the participants’ legal guardians/next of kin in accordance with the national legislation and institutional requirements.

## Author contributions

AV: Conceptualization, Formal analysis, Investigation, Methodology, Validation, Writing – original draft. CG: Conceptualization, Funding acquisition, Resources, Supervision and Writing – review & editing.

## Footnotes

^1^ https://gatk.broadinstitute.org/hc/en-us/articles/360035894711-About-the-GATK-Best-Practices

## Supplementary material

SUPPLEMENTARY FIGURE 1 PWMs for upstream promoter variants showing affinity of regulatory protein. **(A)** variant (rs5772642) **(B)** upstream variant on RABGEF1 (rs1882655) **(C)** upstream variant of CBRN (rs1672753).

SUPPLEMENTARY FIGURE 2 Homozygosity mapping in family 2. **(A)** Pedigree of Family 2 (AD002). **(B)** Visualizing the distribution of homozygous regions in the genome of AD002 case. **(C)** Table listing the identified homozygous regions.

SUPPLEMENTARY FIGURE 3 Homozygosity mapping in family 3. **(A)** Pedigree of Family 3 (AD003). **(B)** Visualizing the distribution of homozygous regions in the genome of AD003 case. **(C)** Table listing the identified homozygous regions.

SUPPLEMENTARY FIGURE 4 Homozygosity mapping in family 4. **(A)** Pedigree of Family 4 (AD004). **(B)** Visualizing the distribution of homozygous regions in the genome of AD004 case. **(C)** Table listing the identified homozygous regions.

SUPPLEMENTARY FIGURE 5 Homozygosity mapping in family 5. **(A)** Pedigree of Family 5 (AD005). **(B)** Visualizing the distribution of homozygous regions in the genome of AD005 case. **(C)** Table listing the identified homozygous regions.

SUPPLEMENTARY FIGURE 6 Homozygosity mapping between SUDs cases and controls. **(A)** Visualizing the distribution of homozygous regions in the genomes of SUDs cases. **(B)** Distribution of homozygous regions across chromosomes. **(C)** Table listing the identified homozygous regions.

SUPPLEMENTARY FIGURE 7 Genes identified via upstream promoter variant analysis is seen juxtaposed in neurotransmitter-neuropeptide axis involved in dopamine regulation. **(A)** NEK9, **(B)** PRRX1, **(C)** PRPF4B, **(D)** CELA2A, **(E)** RABGEF1 and **(F)** CRBN.

SUPPLEMENTARY FIGURE 8 IPA analysis shows miRNAs 548U and 188 under the regulation of immediate early response genes, JUN, and MYC.
