# Supplementary figures and images for: Coordination among frequent genetic variants imparts substance use susceptibility and pathogenesis
Source: Front Neurosci. 2024 Apr 10;18:1332419. doi: 10.3389/fnins.2024.1332419 (PMC11041639; doi:10.3389/fnins.2024.1332419)

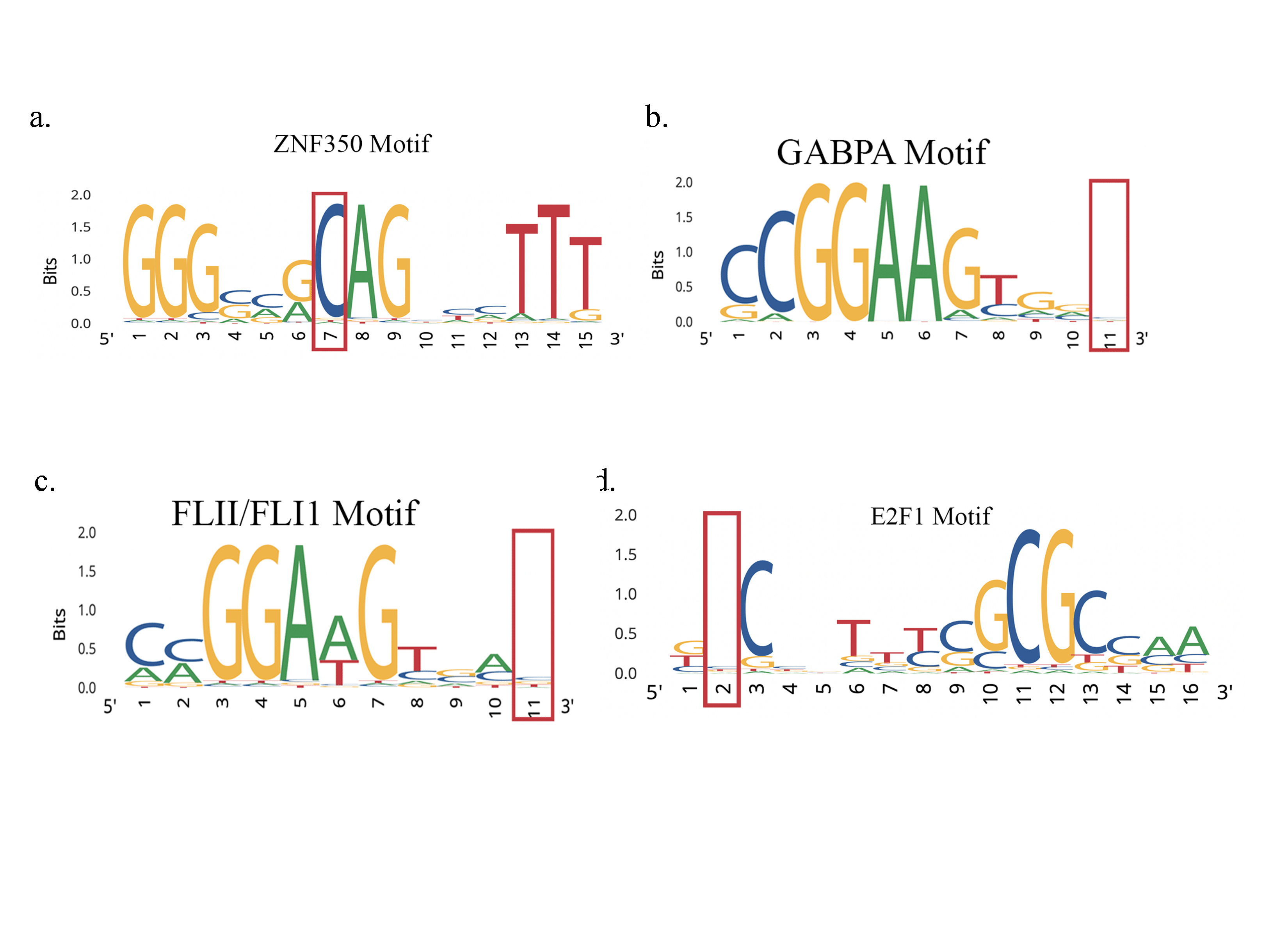

Supplement: SUPPLEMENTARY FIGURE 1 — PWMs for upstream promoter variants showing affinity of regulatory protein. (A) variant (rs5772642) (B) upstream variant on RABGEF1 (rs1882655) (C) upstream variant of CBRN (rs1672753). [file Image_1.JPEG]

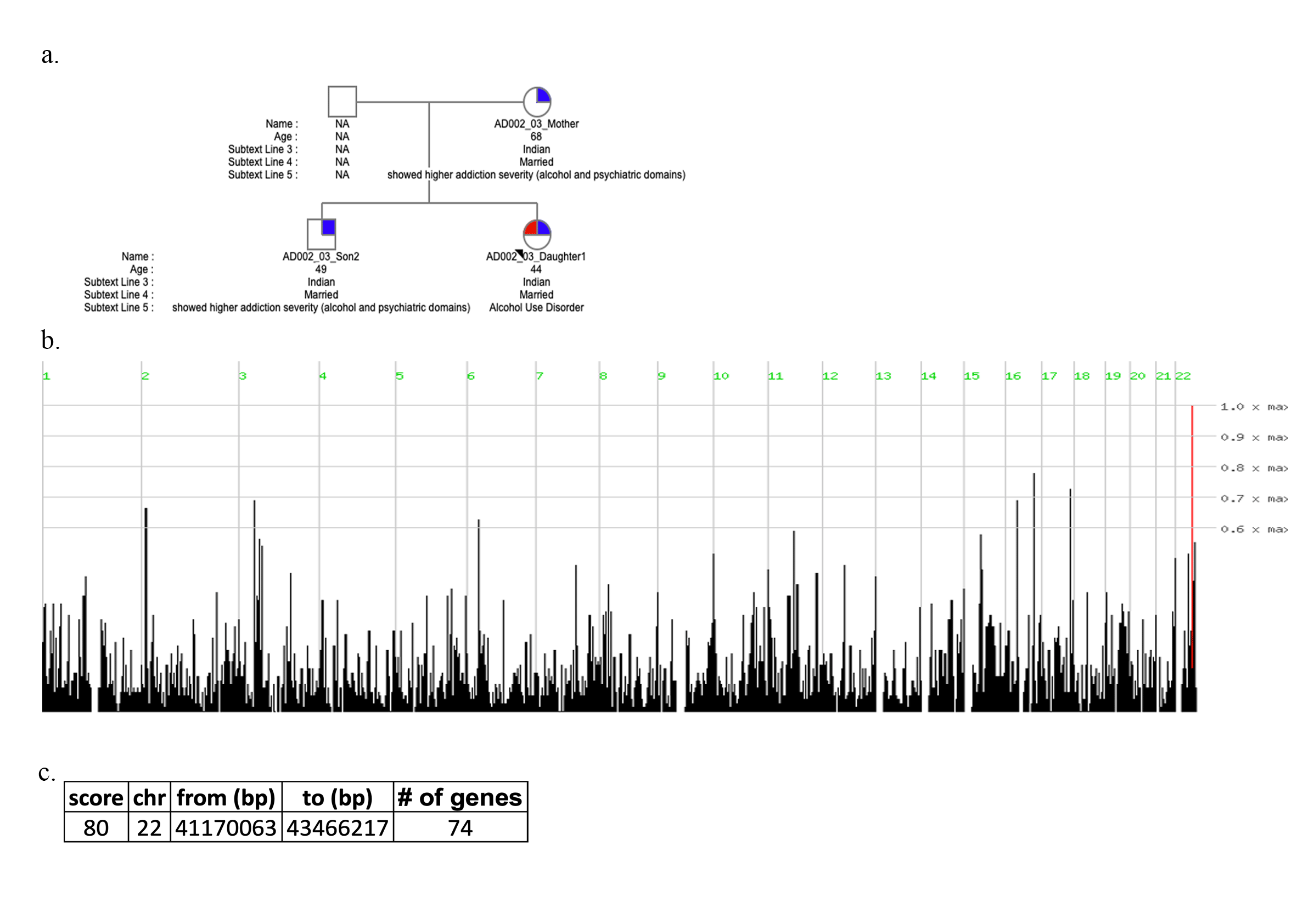

Supplement: SUPPLEMENTARY FIGURE 2 — Homozygosity mapping in family 2. (A) Pedigree of Family 2 (AD002). (B) Visualizing the distribution of homozygous regions in the genome of AD002 case. (C) Table listing the identified homozygous regions. [file Image_2.JPEG]

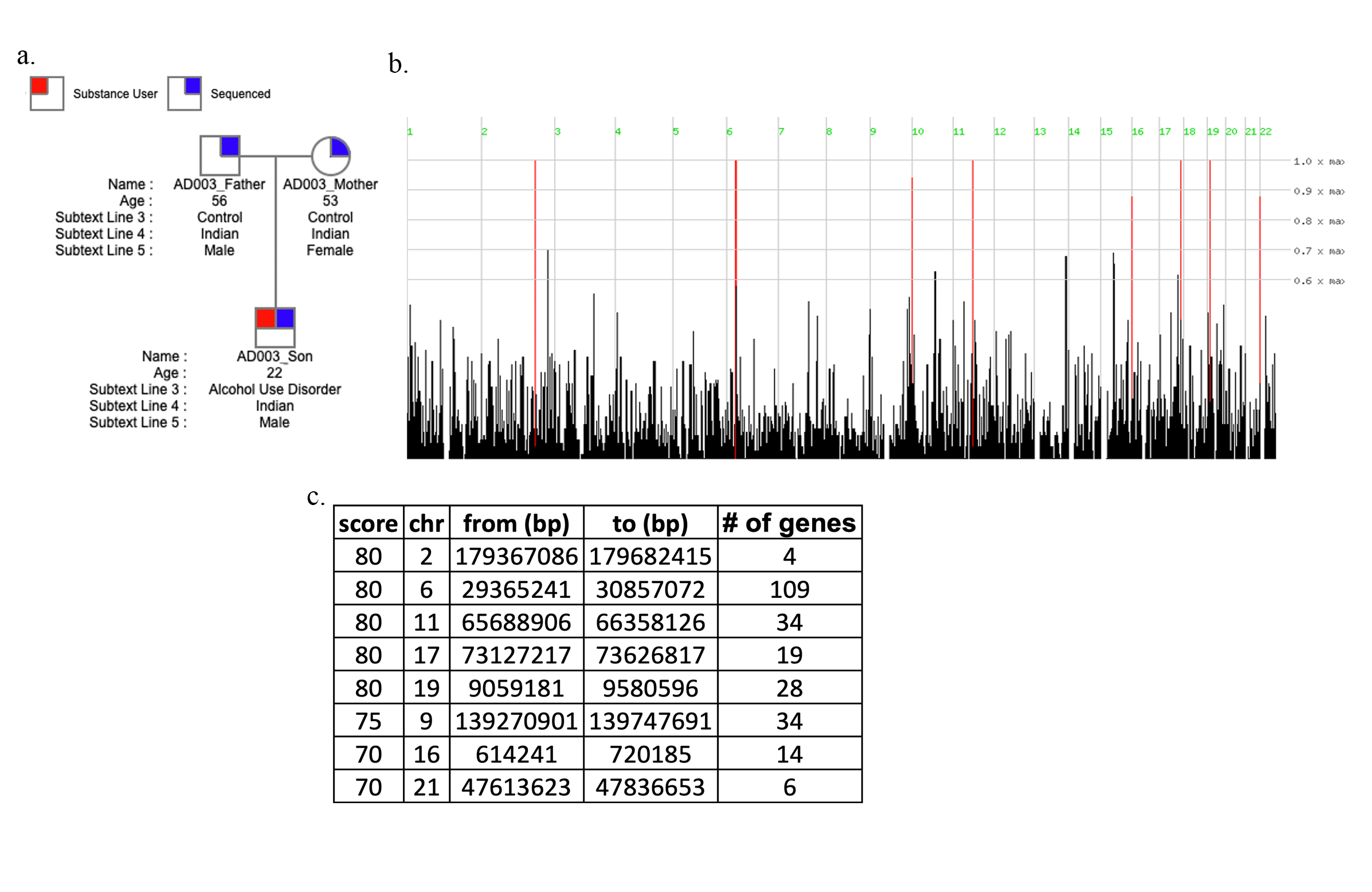

Supplement: SUPPLEMENTARY FIGURE 3 — Homozygosity mapping in family 3. (A) Pedigree of Family 3 (AD003). (B) Visualizing the distribution of homozygous regions in the genome of AD003 case. (C) Table listing the identified homozygous regions. [file Image_3.JPEG]

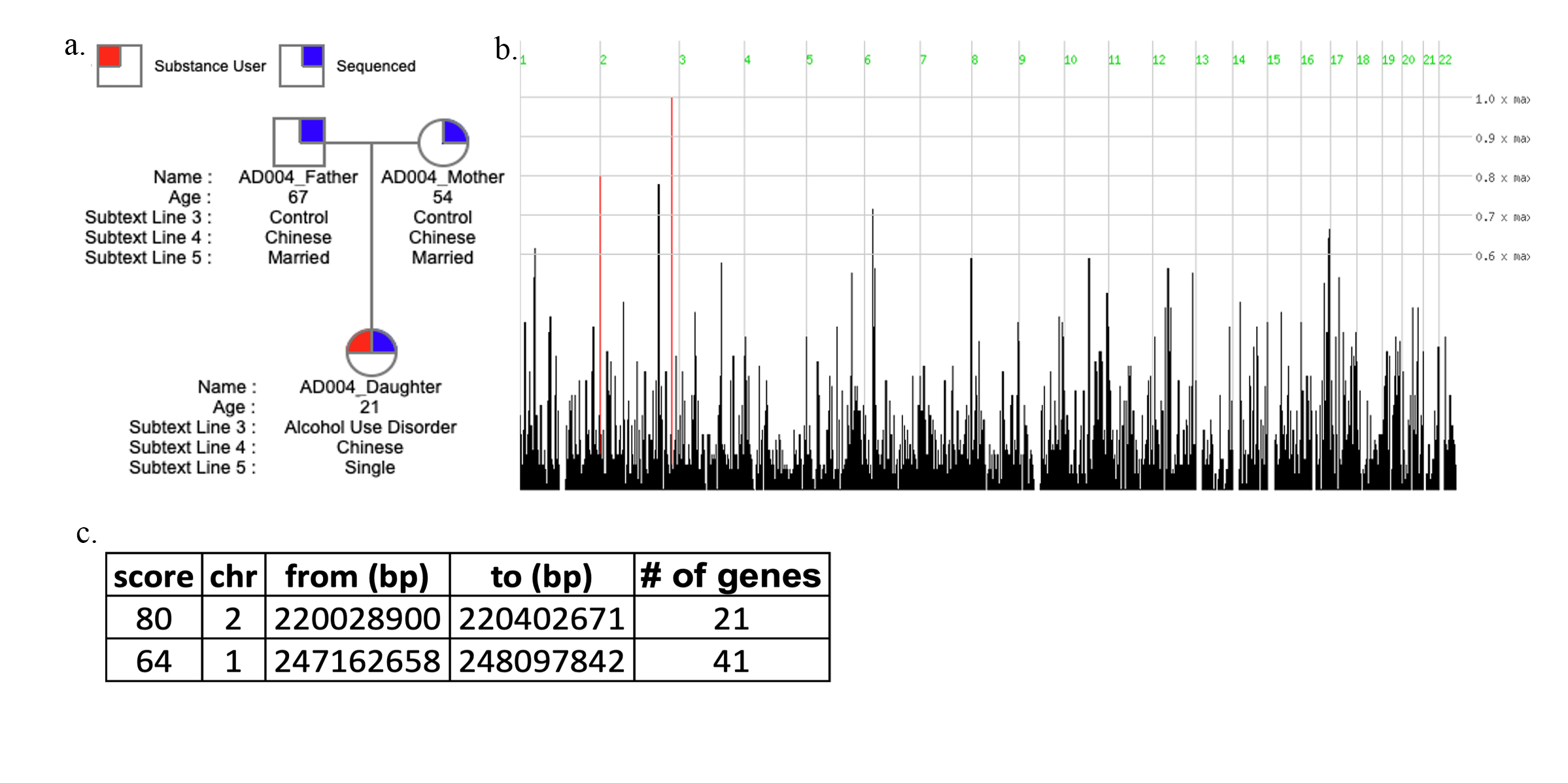

Supplement: SUPPLEMENTARY FIGURE 4 — Homozygosity mapping in family 4. (A) Pedigree of Family 4 (AD004). (B) Visualizing the distribution of homozygous regions in the genome of AD004 case. (C) Table listing the identified homozygous regions. [file Image_4.JPEG]

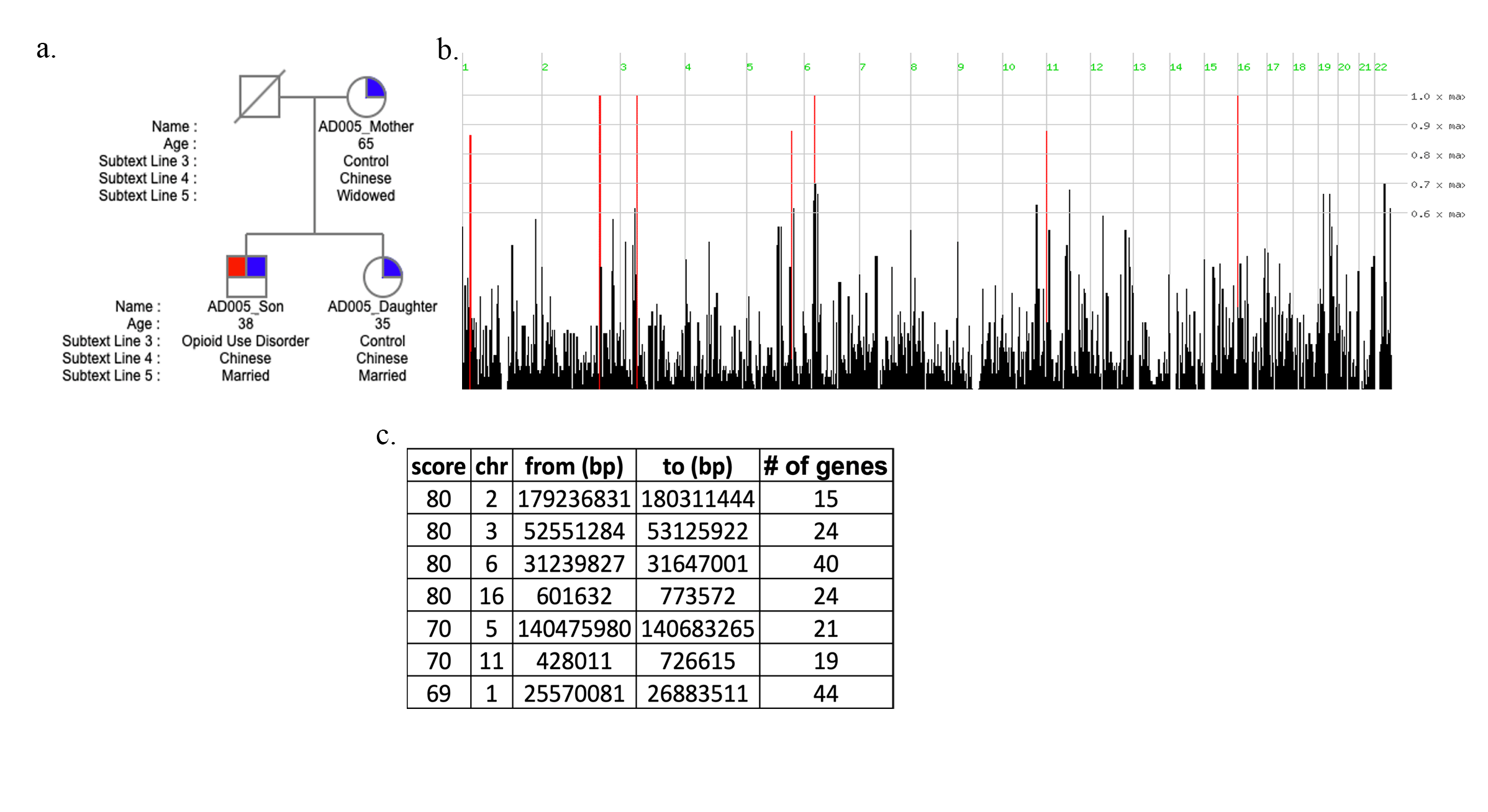

Supplement: SUPPLEMENTARY FIGURE 5 — Homozygosity mapping in family 5. (A) Pedigree of Family 5 (AD005). (B) Visualizing the distribution of homozygous regions in the genome of AD005 case. (C) Table listing the identified homozygous regions. [file Image_5.JPEG]

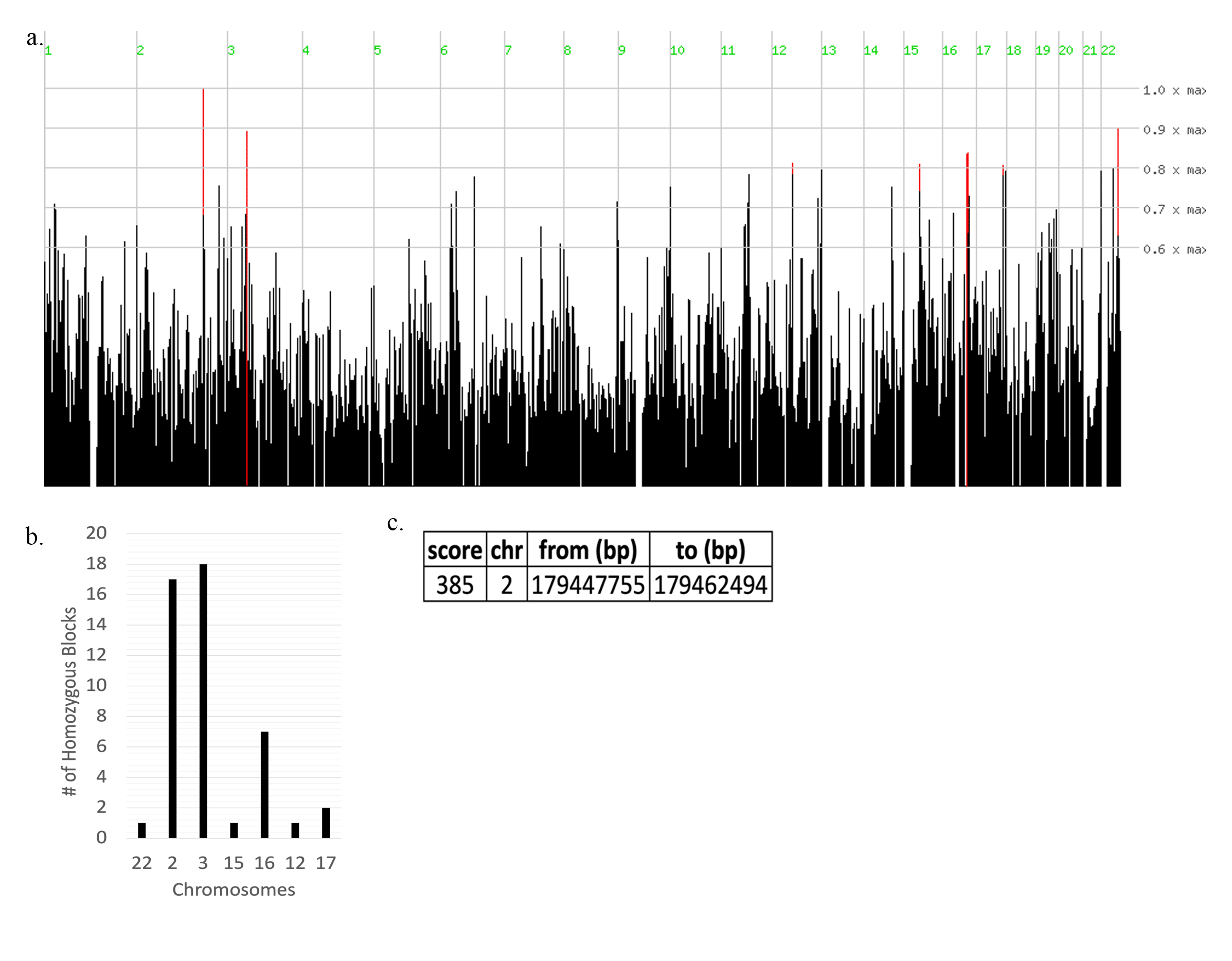

Supplement: SUPPLEMENTARY FIGURE 6 — Homozygosity mapping between SUDs cases and controls. (A) Visualizing the distribution of homozygous regions in the genomes of SUDs cases. (B) Distribution of homozygous regions across chromosomes. (C) Table listing the identified homozygous regions. [file Image_6.JPEG]

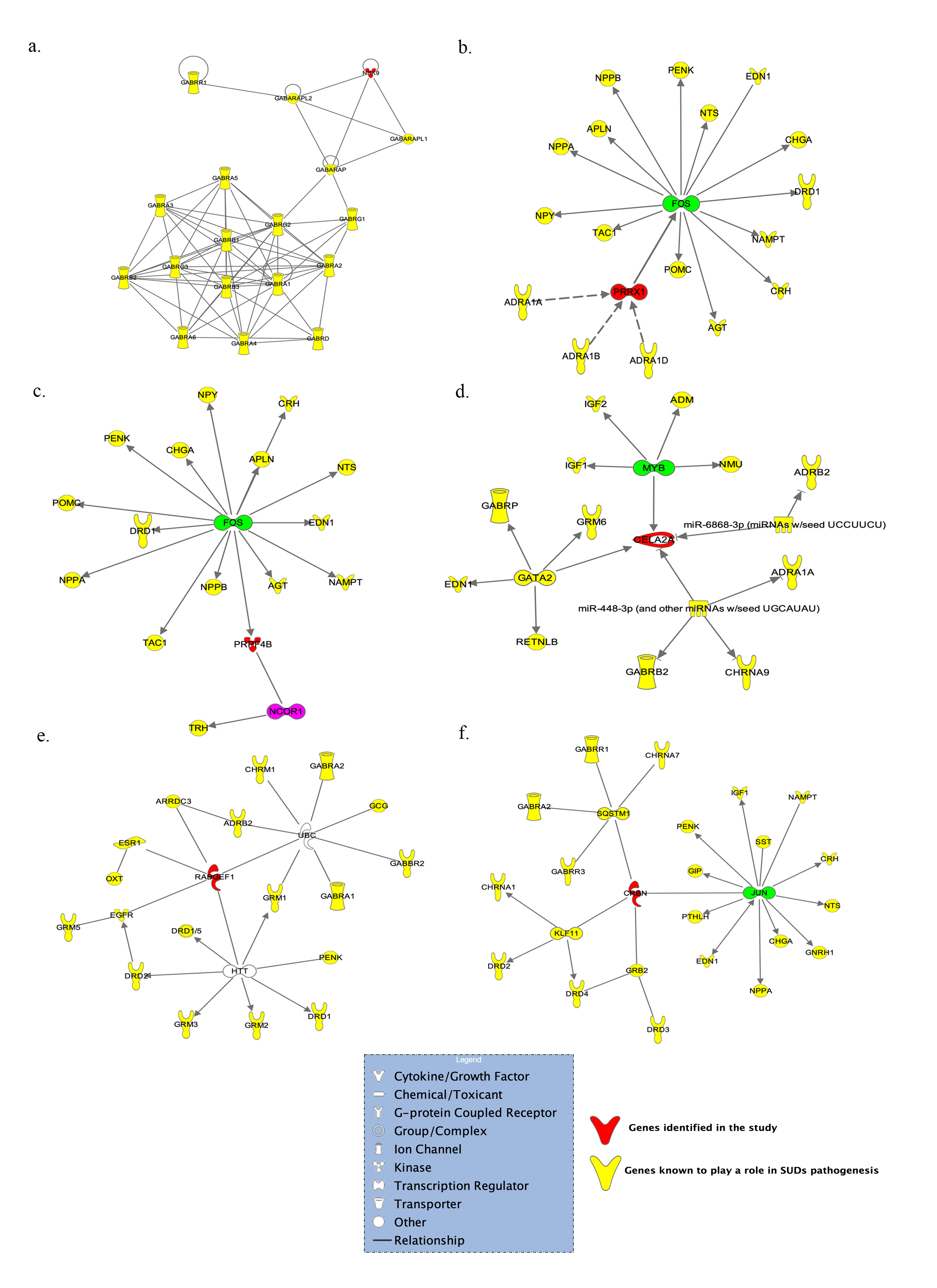

Supplement: SUPPLEMENTARY FIGURE 7 — Genes identified via upstream promoter variant analysis is seen juxtaposed in neurotransmitter-neuropeptide axis involved in dopamine regulation. (A) NEK9, (B) PRRX1, (C) PRPF4B, (D) CELA2A, (E) RABGEF1 and (F) CRBN. [file Image_7.JPEG]

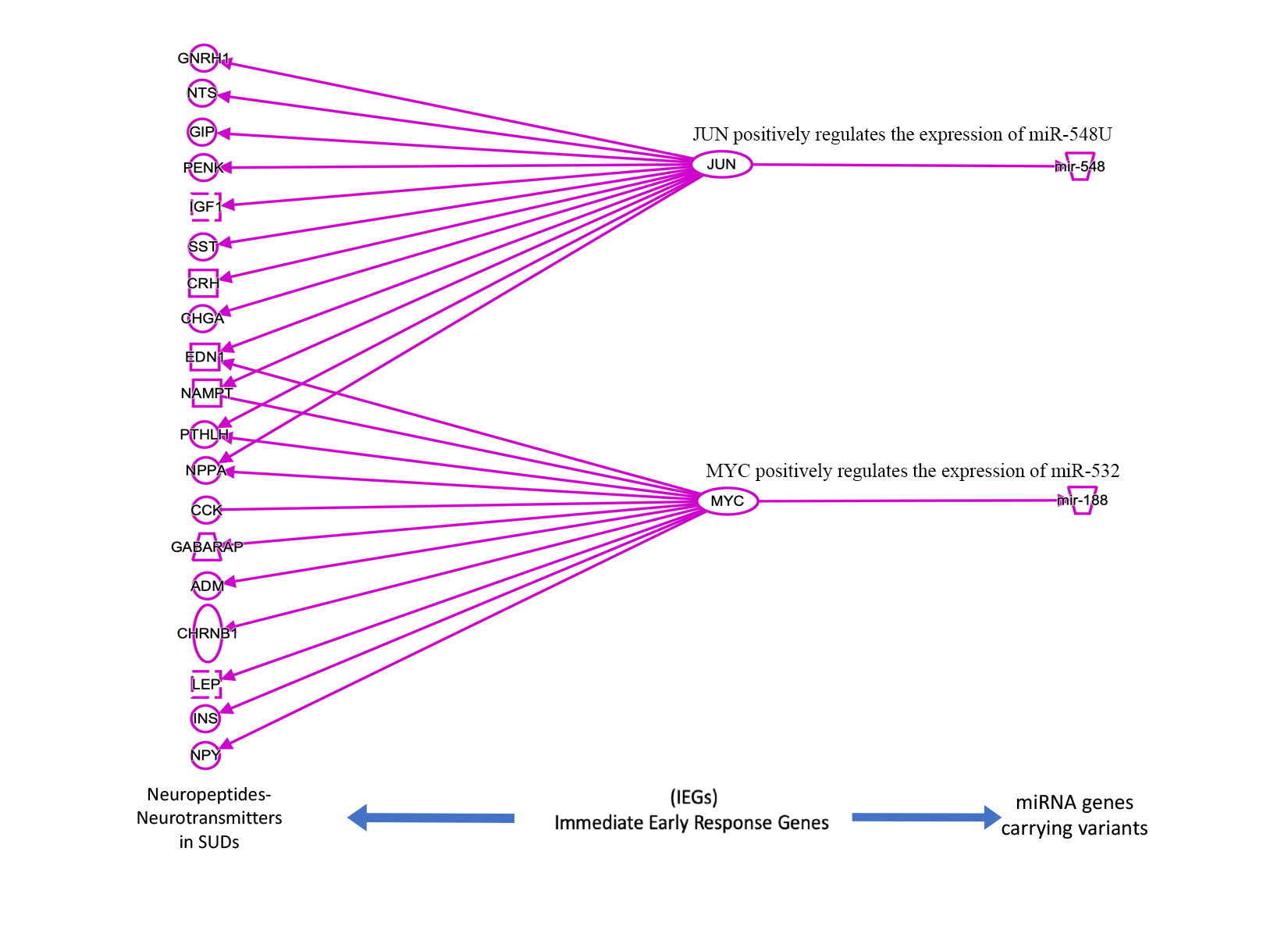

Supplement: SUPPLEMENTARY FIGURE 8 — IPA analysis shows miRNAs 548U and 188 under the regulation of immediate early response genes, JUN, and MYC. [file Image_8.JPEG]
