# Supplementary material for: Coordination among frequent genetic variants imparts substance use susceptibility and pathogenesis
Source: Front Neurosci. 2024 Apr 10;18:1332419. doi: 10.3389/fnins.2024.1332419 (PMC11041639; doi:10.3389/fnins.2024.1332419)
Supplement: Supplementary file 9 [file Table_1.DOCX]

Supplementary Table 1. Socio-demographic features of the trios included in this study. The sample comprised of five trios, with each trio consisting of one subject and two family members, resulting in a total sample size of n=15. All trios included the subject and two parents, except for Trio #2 and #5, which included a subject, one parent, and a sibling. Three of the subjects had a diagnosis of Alcohol Use Disorder, while the remaining two had a diagnosis of Opioid Use Disorder.

| **Trio ID** | **Relationship** | **Diagnosis** | **Age** | **Gender** | **Ethnicity** |
| --- | --- | --- | --- | --- | --- |
| Trio 1 | Proband | Opioid Use Disorder | 32 | Male | Malay |
|  | Mother | - | 60 | Female | Malay |
|  | Father | - | 62 | Male | Malay |
| Trio 2 | Proband | Alcohol Use Disorder | 44 | Female | Indian |
|  | Mother | - | 68 | Female | Indian |
|  | Sibling | - | 49 | Male | Indian |
| Trio 3 | Proband | Alcohol Use Disorder | 22 | Male | Indian |
|  | Mother | - | 53 | Female | Indian |
|  | Father | - | 56 | Male | Indian |
| Trio 4 | Proband | Alcohol Use Disorder | 21 | Female | Chinese |
|  | Mother | - | 54 | Female | Indian |
|  | Father | - | 67 | Male | Chinese |
| Trio 5 | Proband | Opioid Use Disorder | 38 | Male | Chinese |
|  | Mother | - | 65 | Female | Chinese |
|  | Sibling | - | 35 | Female | Chinese |
