# Supplementary material for: Coordination among frequent genetic variants imparts substance use susceptibility and pathogenesis
Source: Front Neurosci. 2024 Apr 10;18:1332419. doi: 10.3389/fnins.2024.1332419 (PMC11041639; doi:10.3389/fnins.2024.1332419)
Supplement: Supplementary file 10 [file Table_2.DOCX]

| Family ID | Chr | Start | End | Ref | Alt | Location | Gene | Variant Type | AA Change | Genotype |
| --- | --- | --- | --- | --- | --- | --- | --- | --- | --- | --- |
| 1 | chr5 | 96315250 | 96315250 | A | - | exonic | LNPEP | frameshift deletion | NM_005575:exon2:c.428delA:p.K143fs  NM_175920:exon2:c.386delA:p.K129fs | het |
| 1 | chr5 | 96315249 | 96315250 | AA | - | exonic | LNPEP | frameshift deletion | NM_005575:exon2:c.427_428del:p.K143fs  NM_175920:exon2:c.385_386del:p.K129fs | het |
| 1 | chr12 | 57593722 | 57593722 | G | - | exonic | LRP1 | frameshift deletion | NM_002332:exon62:c.9928delG:p.G3310fs | het |
| 1 | chr12 | 57593721 | 57593722 | CG | - | exonic | LRP1 | frameshift deletion | NM_002332:exon62:c.9927_9928del:p.P3309fs | het |
| 5 | chr17 | 59485733 | 59485734 | GC | - | exonic | TBX2 | frameshift deletion | NM_005994:exon7:c.2005_2006del:p.A669fs | het |
| 5 | chr17 | 59485734 | 59485734 | C | - | exonic | TBX2 | frameshift deletion | NM_005994:exon7:c.2006delC:p.A669fs | het |

Supplementary Table 2. List of variants found to be compound heterozygous in all the 5 SUDs probands.
