# Supplementary material for: Coordination among frequent genetic variants imparts substance use susceptibility and pathogenesis
Source: Front Neurosci. 2024 Apr 10;18:1332419. doi: 10.3389/fnins.2024.1332419 (PMC11041639; doi:10.3389/fnins.2024.1332419)
Supplement: Supplementary file 12 [file Table_4.DOCX]

Supplementary Table 4: List of variants obtained in 5 probands by genotyping variants from GWAS of SUDs.

| **Family ID** | **Chr** | **Start** | **End** | **Ref** | **Alt** | **Location** | **Gene** | **Variant Type** | **AA Change** | **dbSNP** | **ClinVar DIS** | **Zygosity** |
| --- | --- | --- | --- | --- | --- | --- | --- | --- | --- | --- | --- | --- |
| 1 | chr1 | 46870761 | 46870761 | C | A | exonic | FAAH | nonsynonymous SNV | exon3:c.C385A:p.P129T | rs324420 | Susceptibility to polysubstance abuse | het |
| 1 | chr3 | 133494354 | 133494354 | C | T | exonic | TF | nonsynonymous SNV | exon15:c.C1765T:p.P589S | rs1049296 | Alcohol Consumption | het |
| 1 | chr4 | 100239319 | 100239319 | T | C | exonic | ADH1B | nonsynonymous SNV | exon3:c.A143G:p.H48 | rs1229984 | Alcohol dependence | het |
| 2 | chr1 | 46870761 | 46870761 | C | A | exonic | FAAH | nonsynonymous SNV | exon3:c.C385A:p.P129T | rs324420 | Susceptibility to polysubstance abuse | het |
| 2 | chr4 | 100239319 | 100239319 | T | C | exonic | ADH1B | nonsynonymous SNV | exon3:c.A143G:p.H48R | rs1229984 | Alcohol dependence | hom |
| 2 | chr6 | 154360797 | 154360797 | A | G | exonic | OPRM1 | nonsynonymous SNV | exon1:c.A118G:p.N40D | rs1799971 | Opioid_dependence | het |
| 3 | chr1 | 46870761 | 46870761 | C | A | exonic | FAAH | nonsynonymous SNV | exon3:c.C385A:p.P129T | rs324420 | Susceptibility to polysubstance abuse | het |
| 3 | chr4 | 100239319 | 100239319 | T | C | exonic | ADH1B | nonsynonymous SNV | exon3:c.A143G:p.H48R | rs1229984 | Alcohol dependence | hom |
| 3 | chr4 | 100260789 | 100260789 | T | C | exonic | ADH1C | SNV | chr4:100260789-100260789 | rs698 | Alcohol dependence | hom |
| 3 | chr6 | 154360797 | 154360797 | A | G | exonic | OPRM1 | nonsynonymous SNV | exon1:c.A118G:p.N40D | rs1799971 | Opioid_dependence | het |
| 3 | chr7 | 75211414 | 75211414 | A | G | exonic | HIP1 | synonymous SNV | exon6:c.T519C:p.A173A | rs237238 | Alcoholism (12-month weekly alcohol consumption) | het |
| 3 | chr20 | 61981104 | 61981104 | C | T | exonic | CHRNA4 | synonymous SNV | exon5:c.G1659A:p.A553A | rs1044397 | Nicotine addiction | het |
| 3 | chr20 | 61981134 | 61981134 | G | A | exonic | CHRNA4 | synonymous SNV | exon5:c.C1629T:p.S543S | rs1044396 | Nicotine addiction | het |
| 4 | chr1 | 46870761 | 46870761 | C | A | exonic | FAAH | nonsynonymous SNV | exon3:c.C385A:p.P129T | rs324420 | Susceptibility to polysubstance abuse | het |
| 4 | chr4 | 100239319 | 100239319 | T | C | exonic | ADH1B | nonsynonymous SNV | exon3:c.A143G:p.H48R | rs1229984 | Alcohol dependence | het |
| 4 | chr20 | 61981104 | 61981104 | C | T | exonic | CHRNA4 | synonymous SNV | exon5:c.G1659A:p.A553A | rs1044397 | Nicotine addiction | het |
| 4 | chr20 | 61981134 | 61981134 | G | A | exonic | CHRNA4 | synonymous SNV | exon5:c.C1629T:p.S543S | rs1044396 | Nicotine addiction | het |
| 4 | chr9 | 101340316 | 101340316 | T | C | exonic | GABBR2 | synonymous SNV | exon2:c.A360G:p.A120A | rs3750344 | Susceptibility to tobacco addiction | het |
| 5 | chr1 | 46870761 | 46870761 | C | A | exonic | FAAH | nonsynonymous SNV | exon3:c.C385A:p.P129T | rs324420 | Susceptibility to polysubstance abuse | het |
| 5 | chr20 | 61443716 | 61443716 | G | A | exonic | OGFR | nonsynonymous SNV | exon7:c.G749A:p.R250Q | rs41309371 | . | het |
