# Supplementary material for: Coordination among frequent genetic variants imparts substance use susceptibility and pathogenesis
Source: Front Neurosci. 2024 Apr 10;18:1332419. doi: 10.3389/fnins.2024.1332419 (PMC11041639; doi:10.3389/fnins.2024.1332419)
Supplement: Supplementary file 13 [file Table_5.DOCX]

Supplementary Table 5: Variants identified in upstream and exonic regions of miRNA genes from 5 SUDs probands.

| Chr | Start | End | Ref | Alt | Location | Gene | 1000G SAS | ExAC SAS | dbSNP | Zygosity |
| --- | --- | --- | --- | --- | --- | --- | --- | --- | --- | --- |
| chr6 | 57254955 | 57254955 | G | A | ncRNA_exonic | MIR548U | . | 0.5 | rs2894842 | het |
| chrX | 49767832 | 49767832 | A | G | ncRNA_exonic | MIR532 | 1 | 1 | rs456615 | hom |
| chrX | 49767835 | 49767835 | A | G | ncRNA_exonic | MIR532 | 1 | 1 | rs456617 | hom |
